# Tensor decomposition-based unsupervised feature extraction identifies candidate genes that induce post-traumatic stress disorder-mediated heart diseases

**DOI:** 10.1186/s12920-017-0302-1

**Published:** 2017-12-21

**Authors:** Y.-H. Taguchi

**Affiliations:** Department of Physics, Chuo University, 1-13-27 Kasuga, Bunkyo-ku, Tokyo, 112-8551 Japan

**Keywords:** Tensor decomposition, Feature extraction, Post-traumatic stress disorder, Heart disease

## Abstract

**Background:**

Although post-traumatic stress disorder (PTSD) is primarily a mental disorder, it can cause additional symptoms that do not seem to be directly related to the central nervous system, which PTSD is assumed to directly affect. PTSD-mediated heart diseases are some of such secondary disorders. In spite of the significant correlations between PTSD and heart diseases, spatial separation between the heart and brain (where PTSD is primarily active) prevents researchers from elucidating the mechanisms that bridge the two disorders. Our purpose was to identify genes linking PTSD and heart diseases.

**Methods:**

In this study, gene expression profiles of various murine tissues observed under various types of stress or without stress were analyzed in an integrated manner using tensor decomposition (TD).

**Results:**

Based upon the obtained features, ∼ 400 genes were identified as candidate genes that may mediate heart diseases associated with PTSD. Various gene enrichment analyses supported biological reliability of the identified genes. Ten genes encoding protein-, DNA-, or mRNA-interacting proteins—ILF2, ILF3, ESR1, ESR2, RAD21, HTT, ATF2, NR3C1, TP53, and TP63—were found to be likely to regulate expression of most of these ∼ 400 genes and therefore are candidate primary genes that cause PTSD-mediated heart diseases. Approximately 400 genes in the heart were also found to be strongly affected by various drugs whose known adverse effects are related to heart diseases and/or fear memory conditioning; these data support the reliability of our findings.

**Conclusions:**

TD-based unsupervised feature extraction turned out to be a useful method for gene selection and successfully identified possible genes causing PTSD-mediated heart diseases.

**Electronic supplementary material:**

The online version of this article (doi:10.1186/s12920-017-0302-1) contains supplementary material, which is available to authorized users.

## Background

Post-traumatic stress disorder (PTSD) [1] is primarily a mental illness caused by stressors. Nevertheless, PTSD can cause additional symptoms apparently not directly related to the central nervous system. PTSD-mediated heart diseases are some of such examples [2]. Although it is believed that PTSD highly correlates with heart failure [3], the mechanisms by which PTSD mediates heart failure are still unclear. Because a study on twins revealed a strong correlation between PTSD and heart diseases [4], the genomic factors are believed to link the two disorders. Therefore, in this study, tensor decomposition (TD)-based unsupervised feature extraction (FE)—which is the extension of a recently proposed principal component analysis-based unsupervised FE that has been successfully applied to various bioinformatics problems [5–22]—was used for various gene expression profiles of murine tissues with the aim to find genes coexpressive or differentially expressed between stressful and unstressful conditions in the brain and heart. As shown in the text below, we identified approximately 400 genes using TD-derived features, and these genes are strongly related to neurodegenerative diseases as well as cardiac-muscle aberrations. Furthermore, the top 10 genes encoding protein-, DNA-, or mRNA-interacting proteins were identified as those governing expression of these ∼ 400 genes and are possible therapeutic targets in PTSD-mediated heart diseases according to other reports.

## Methods

### Gene expression

The gene expression profiles used in this study were downloaded from the Gene Expression Omnibus (GEO) database (GEO ID GSE68077). The file “GSE68077_series_matrix.txt” that is available as “Series Matrix File(s)” was downloaded. Probes whose names start with “EA” were removed. The gene expression profile was standardized (i.e., means and variances in each sample are 0 and 1, respectively).

Gene expression profiles were formatted as a tensor, $x_{i,j_{1},j_{2},j_{3},j_{4}}$, of the *i*th probe, subjected to *j*_1_th treatment (*j*_1_=1: control, *j*_1_=2: treated [stress-exposed] samples), in the *j*_2_th tissue [ *j*_2_=1: amygdala (AY), *j*_2_=2: hippocampus (HC), *j*_2_=3: medial prefrontal cortex (MPFC), *j*_2_=4: septal nucleus (SE), *j*_2_=5: striatum (ST), *j*_2_=6: ventral striatum (VS), *j*_2_=7: blood, *j*_2_=8: heart, *j*_2_=9: hemibrain, *j*_2_=10: spleen], with the *j*_3_th stress duration (*j*_3_=1: 10 days, *j*_3_=2: five days) and *j*_4_th rest period after application of stress (*j*_4_=1: 1.5 weeks, *j*_4_=2: 24 hours, *j*_4_=3: 6 weeks). Zero values were assigned to missing observations (e.g., measurements at 6 weeks after a 5-day period of stress are not available).

### TD-based unsupervised FE

To perform gene selection using gene singular value vectors, $x_{\ell _{1},i_{1}}$ for synthetic data and $x_{\ell _{5},i}$ for real gene expression profiles, we have to decide which singular value vectors are used for the selection. If we denote this set of gene singular value vectors as *Ω*, then a *P*-value, $P_{i_{1}}$, for synthetic data and *P*_*i*_ for real gene expression profiles, is assigned to each gene by assuming that the singular value vectors of genes obey a *χ* ^2^ distribution,

$$P_{i_{1}} = P \left [ > \sum_{\ell_{5} \in \Omega} \left(\frac{x_{\ell_{1},i_{1}}}{\sigma_{\ell_{1}}}\right)^{2}\right] $$

for synthetic datasets and

$$P_{i} = P \left [ > \sum_{\ell_{5} \in \Omega} \left(\frac{x_{\ell_{5},i}}{\sigma_{\ell_{5}}}\right)^{2}\right] $$

for real gene expression profiles, where $\sigma _{\ell _{1}}$ and $\sigma _{\ell _{5}}$ are the standard deviation of the *ℓ*_1_th and *ℓ*_5_th singular value vectors ($x_{\ell _{1},i_{1}}$ and $x_{\ell _{5},i}$), whereas *P*[>*x*] is the *P*-value for the hypothesis that the argument is greater than *x*, assuming a *χ* ^2^ distribution. After that, genes associated with adjusted *P*-values less than 0.01, 0.05, and 0.1 for the synthetic dataset and 0.01 for real gene expression profiles were selected, respectively.

### Computation of adjusted *P*-values

The adjusted *P*-values were computed either by the p.adjust function in R [23] with the “BH” (Benjamini–Hochberg) option or the fdrtool in R [23] with the statistic=“pvalue” option. The AUC was computed by means of the colAUC function in the caTools package in R [23].

### The synthetic dataset

**Fig. 1:**
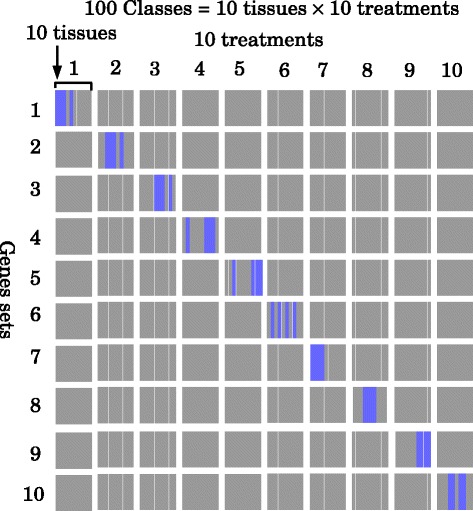
The gene expression pattern of 10 gene sets. Each of which includes 100 genes from synthetic data (thus, 1000 of the total of 30,000 genes are being considered). The remaining 29,000 genes do not have any class specificity. Blue squares represent classes where the genes in each gene set are expressive. Ten tissues are assumed to be treated in 10 distinct ways. Thus, in total, there are 100 classes

This dataset is composed of a 30,000×10×10 tensor, $x_{i_{1},i_{2},i_{3}},1 \le i_{1} \le 30,000, 1 \le i_{2},i_{3}\le 10$. The first (*i*_1_), second (*i*_2_), and third (*i*_3_) modes represent genes, tissues, and treatments, respectively. For each of 10 treatments, 100 genes were expressed in four out of 10 tissues. The first gene through the 100th gene are expressive during the first treatment, the 101st to 200th genes are expressive during the second treatment, and so on. If a combination of a gene set and class falls into a blue filled square in Fig. 1 (e.g., the second gene set in the third class), then $x_{i_{1},i_{2},i_{3}}$ obeys a Gaussian distribution of mean 4 and variance 1; otherwise, the mean is assumed to be zero. *x*_*ij*_ is standardized within each sample as well.

### Enrichment analyses

These analyses were conducted by means of g:profiler r1622_e84_eg31 [24] and Enrichr [25]. All probe IDs were converted to gene symbols before uploading to the servers. For g:profiler, all the genes included in the microarray were uploaded as a background.

### Enrichment analysis of MSigDB

A total of 457 gene symbols were uploaded to http://software.broadinstitute.org/gsea/msigdb/annotate.jsp(registration and login are needed). The option “C2: curated gene sets” was selected.

### Clustering analysis

For synthetic data, two clustering methods were used. The first is hierarchical clustering (Ward method) using the Euclidean distance between the first gene’s and 10th gene’s singular value vectors, $x_{\ell _{1},i_{1}},1 \le \ell _{1} \le 10$, as the distance. Then, the generated trees were partitioned into 11 clusters. The Ward method was implemented as the hclust function in R [23] with the method =“ward” option. Partitioning was performed using the cutree function in R using k =11 option (the number of clusters is 11). The second one is a Gaussian mixture, which was carried out by the Mclust function in the mclust [26] package in R [23] with the G =1:11 option (assuming 1 to 11 clusters).

## Results

### TD applied to tensors of gene expression profiles

In this study, gene expression profiles were regarded as tensors. Gene expression profiles were analyzed in various tissues including the heart and brain, under various conditions (stressful or unstressful), with various periods of stress and rest time after application of a stressor. These datasets were naturally regarded as a tensor, $x_{i,j_{1},\ldots,j_{m}}$, where *i* stands for genes and *j*_*k*_,*k*=1,…,*m* denotes various tissues as well as experimental conditions. To reduce the number of degrees of freedom, tensors can be decomposed to smaller tensors, vectors, or matrices. Although there are multiple implementations, higher-order singular value decomposition [27] (HOSVD) was employed in the present study, and a tensor was decomposed as $x_{i,j_{1},\ldots,j_{m}} = \sum _{\ell _{1},\ldots,\ell _{m+1}} G (\ell _{1}, \ldots, \ell _{m+1}) \cdot x_{\ell _{m+1},i} \prod _{k=1}^{m} x_{\ell _{k},j_{k}}$, where *G* is the core tensor and $x_{\ell _{k},j_{k}}$ and $x_{\ell _{m+1},i}$ are singular value matrices. In this implementation, singular value vectors, $x_{\ell _{k},j_{k}}$ or $x_{\ell _{m+1},j}$, associated with *G* with greater absolute values primarily and correlatively contribute to the original tensor, $x_{i,j_{1},\ldots,j_{m}}$ ($x_{\ell _{k},j_{k}}, k=1,\ldots,m, x_{\ell _{m+1},i}$ are supposed to be orthogonal matrices and thus have the same absolute values and equally contribute to the tensor, whereas only the amount of *G* counts for the contribution).

### Synthetic data

Prior to application to gene expression profiles, TD was applied to a synthetic dataset to demonstrate usefulness of our strategy. In this synthetic dataset, 10 tissues were assumed to be treated with 10 experimental conditions (thus, there were assumed to be 10×10=100 samples). In each experimental condition, in four out of 10 tissues, 100 distinct genes were expressed (Fig. 1). Thus, in total, 1,000 genes were expressive in some of the 10 tissues under some of the conditions tested. The remaining majority of genes (as many as 29,000 because in total 30,000 genes are assumed to exist) were not expressed at all in any tissues under any conditions. The task was to identify separately 10 sets of 100 genes as being coexpressive in four tissues.

**Fig. 2:**
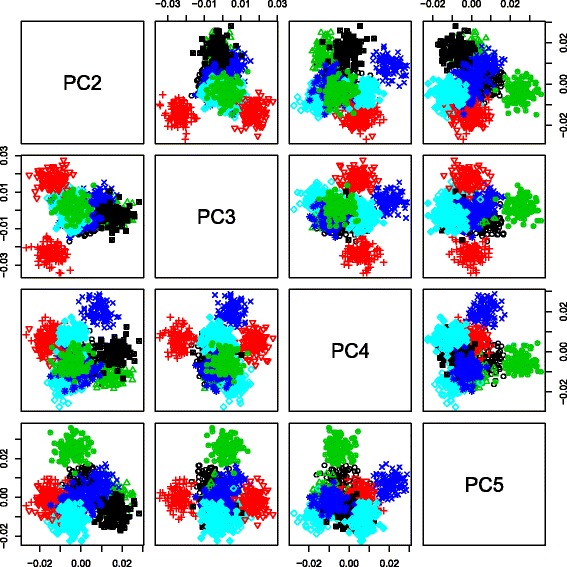
Scatter plots involving the second gene’s through fifth gene’s singular value vectors. $x_{\ell _{1},i_{1}}, 2 \le \ell _{1} \le 5$, of 1,000 genes (1≤*i*_1_≤1000) that belong to one of the 10 gene sets. These 10 gene sets are represented by distinct combinations of colors and symbols. The 29,000 genes not included in any of the 10 gene sets are omitted for clarity

**Fig. 3:**
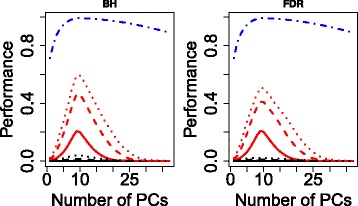
Performance of synthetic data (averaged over 100 trials). BH: Benjamini-Hochberg, FDR: false discovery rate. Red curves: true positive rates, black curves: false positive rates, solid curves: *P*=0.01, dashed curves: *P*=0.05, dotted curves: *P*=0.1, blue dash-and-dot curves: area under the curve (AUC)

Applying TD to the synthetic dataset,

$$x_{i_{1},i_{2},i_{3}} = \sum_{\ell_{1},\ell_{2},\ell_{3}} G(\ell_{1},\ell_{2},\ell_{3}) x_{\ell_{1},i_{1}}x_{\ell_{2},i_{2}}x_{\ell_{3},i_{3}},$$

genes were embedded into the space spanned by the derived gene singular value vectors, $x_{\ell _{1},i_{1}}$. We found that they are strictly clustered coincidently with the 10 presumed clusters (Fig. 2). Although genes identified as outliers by means of these gene singular value vectors, $x_{\ell _{1},i_{1}}$, were extracted, it was obvious that TD-based unsupervised FE successfully identified some of the 1,000 genes with a relatively smaller number of false positives no matter which adjusted *P*-values were employed as threshold values (Fig. 3).

To test quantitatively whether the 10 clusters of the genes were identified correctly, hierarchical clustering as well as mixture Gaussian clustering were performed. Ten gene clusters were found to be identified correctly, and expression patterns were also correct (Table 1 and Additional file 1).

**Table 1:** Clustering of genes identified by TD-based unsupervised FE for synthetic data

	1	2	3	4	5	6	7	8	9	10	11
	Mclust										
1	80	0	0	0	0	0	0	0	0	0	5
2	0	71	0	0	0	0	0	0	0	0	5
3	0	0	90	0	0	0	0	0	0	0	1
4	0	0	0	65	0	0	0	0	0	0	1
5	0	0	0	0	69	0	0	0	0	0	2
6	0	0	0	0	0	00	0	0	0	0	16
7	0	0	0	0	0	66	0	0	0	0	2
8	0	0	0	0	0	0	66	0	0	0	5
9	0	0	0	0	0	0	0	77	0	0	4
10	0	0	0	0	0	0	0	0	74	0	3
11	0	0	0	0	0	0	0	0	0	81	11
	Ward										
1	81	0	0	0	0	0	0	0	0	0	5
2	0	71	0	0	0	0	0	0	0	0	4
3	0	0	90	0	0	0	0	0	0	0	3
4	0	0	0	65	0	0	0	0	0	0	1
5	0	0	0	0	69	0	0	0	0	0	8
6	0	0	0	0	0	66	0	0	0	0	4
7	0	0	0	0	0	0	66	0	0	0	8
8	0	0	0	0	0	0	0	77	0	0	5
9	0	0	0	0	0	0	0	0	64	0	4
10	0	0	0	0	0	0	0	0	0	35	3
11	0	0	0	0	0	0	0	0	0	46	10

Rows: gene sets (the first to the tenth are the gene sets to which the first 1000 genes are likely to belong, and the 11th is the gene set to which the remaining 29,000 genes are likely to belong), columns: clustering

In the next subsection, TD-based unsupervised FE is applied to real gene expression profiles.

### Real gene expression profiles

**Fig. 4:**
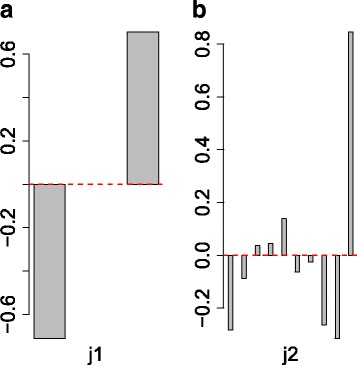
Singular value vectors employed **a** The second control-related or treatment-related singular value vector, $x_{\ell _{1}=2,j_{1}}$. Control: *j*_1_=1, and treatment (stress): *j*_1_=2. **b** The fourth tissue singular value vector, $x_{\ell _{2}=4,j_{2}}$, AY: *j*_2_=1, HC: *j*_2_=2, heart: *j*_2_=8, hemibrain: *j*_2_=9, and spleen: *j*_2_=10. Other tissue singular value vectors, *ℓ*_2_≠4, can be found in Additional file 2

In the previous subsection, the usefulness of our strategy was successfully demonstrated on synthetic data. In this subsection, TD-based unsupervised FE is applied to real data, i.e., gene expression profiles [28], which were formatted as a five-mode tensor that contains indices corresponding to genes (*i*) versus tissues (*j*_2_) vs stress duration (*j*_3_) vs rest period after application of stress (*j*_4_) vs control or treatment (*j*_1_) (Table 2). Replicates in each category were averaged within each category before TD was applied. It is definitely a challenging dataset because genes being sought must be differentially expressed between treated and control samples, not in all tissues but in some tissues. HOSVD was applied to the tensor and five singular value matrices, $x_{\ell _{k},j_{k}}, 1\le k \le 4$, and $x_{\ell _{5},i}$, were obtained. Figure 4a shows the second control-related or treatment-related singular value vectors, $x_{\ell _{1}=2,j_{1}}, j_{1}=1,2$. The findings indicate that this expression represents a difference between control (*j*_1_=1) and treated (*j*_1_=2) samples. Next, tissue singular value vectors, $x_{\ell _{2},j_{2}}, 1 \le \ell _{2}, j_{2} \le 10$, were studied (Fig. 4b and Additional file 2). Then, the fourth tissue singular value vector, $x_{\ell _{2}=4,j_{2}}, 1 \le j_{2} \le 10$, was found to show coexpression among amygdala (AY, *j*_2_=1), hippocampus (HC, *j*_2_=1), and heart (*j*_2_=8), which represents the phenotypes of interest. After that, we investigated which gene singular value vectors are associated with the fourth sample as well as the second control-related or treatment-related singular value vectors. Table 3 shows the top-ranked core tensor *G*(*ℓ*_1_=2,*ℓ*_2_=4,*ℓ*_3_,*ℓ*_4_,*ℓ*_5_) with greater absolute values. Then, gene singular values vectors, $x_{\ell _{5} \in (1,4,11),i} $, were identified as being associated with the fourth sample as well as the second control-related or treatment-related singular value vectors. After that, 801 probes (Additional file 3) associated with adjusted *P*-values less than 0.01 were selected as outliers using these three gene singular value vectors.

**Table 2:** Samples used in this study Numbers before/after comma are control/treated samples

stress, days	5		10			5		10	
rest period	24h	1.5 w	24h	6w		24h	1.5 w	24h	6w
AY	3,2	5,4	3,4	3,4	HC	3,5	4,5	5,4	4,5
MPFC	4,5	5,5	3,4	4,4	SE	3,2	2,3	3,3	3,3
ST	5,5	5,5	5,4	4,4	VS	5,5	5,5	3,4	5,4
blood	5,5	5,5	4,5	4,5	heart	5,5	4,5	5,5	5,5
hemibrain	5,5	4,5	5,5	5,5	spleen	5,5	5,5	5,4	5,5

h: hours, w: weeks

AY: amygdala, HC: hippocampus, MPFC: medial prefrontal cortex, SE: septal nucleus, ST: striatum, VS: ventral striatum

**Table 3:** Top-ranked *G*(*ℓ*_1_=2,*ℓ*_2_=4,*ℓ*_3_,*ℓ*_4_,*ℓ*_5_) with greater absolute values

*ℓ*_3_	*ℓ*_4_	*ℓ*_5_	*G*(2,4,*ℓ*_3_,*ℓ*_4_,*ℓ*_5_)
1	1	11	-35.0
1	1	1	-30.8
2	2	1	-30.3
2	3	4	-30.0
2	3	1	28.7
2	2	4	28.5

To determine whether the 801 selected probes are selectively expressive in the AY, HC, and heart as expected, the *t* test was applied to all the 40 combinations of control and treated samples. Then, 13 combinations (Table 4) turned out to have the adjusted *P*-values less than 0.01. Because the AY, HC, and heart are abundantly represented in Table 4, it is obvious that our strategy, TD-based unsupervised FE, successfully identified probes selectively coexpressive in AY, HC, and heart between control and treated samples.

**Table 4:** Thirteen combinations of tissues and experimental conditions where the selected 801 probes are differentially expressed between Stress-exposed and control samples

stress duration	10 days		5 days	
rest period	24 hours	6 weeks	24 hours	1.5 weeks
AY		○		○
HC		○	○	○
MPFC		○		
Heart	○			○
Hemibrain			○	○
Spleen		○	○	○

MPFC: medial prefrontal cortex

### Comparison with other methodologies

In contrast to the success of this strategy, which was applied to a synthetic dataset and real gene expression profiles, other tested methods failed to identify some of the 1000 genes correctly in synthetic data (Additional file 4) and failed to identify some of the significantly differentially expressed genes (Additional file 4).

## Discussion

Biological reliability was evaluated by means of 457 gene symbols (Additional file 5) that are associated with the 801 identified probes, uploaded to g:profiler. Various biological terms were enriched (Additional file 6): 198 Gene Ontology (GO) biological process (BP) terms, 79 GO cellular component (CC) terms, 49 GO molecular function (MF) terms, 7 Kyoto Encyclopedia of Genes and Genomes (KEGG) pathways, and 38 REACTOME pathways. Among them, neurodegenerative-disease-related KEGG pathways were enriched (three categories): “Huntington’s disease,” “Parkinson’s disease,” and “Alzheimer’s disease” as well as one KEGG heart disease-related pathway, “Cardiac muscle contraction”, GO BP terms, “heart contraction” and “cardiac muscle contraction” as well as GO CC term “neuron part” were also identified. Thus, these findings suggest that the identified genes are potentially related to neuronal functions as well as heart anomalies.

To evaluate the relation between the selected genes and PTSD-mediated heart diseases, two biological terms, “nonsense-mediated decay” (NMD; REACTOME) and “SRP-dependent cotranslational protein targeted to membrane” (REACTOME, GO BP), were further analyzed because these two are reported to be specifically related to fear memory and heart failure (see below).

NMD was reported to regulate cardiac myosin-binding protein C mutant levels in cardiomyopathic mice [29]. Arc mRNA is targeted for NMD, and time-tependent expression of Arc and Zif268 after acquisition of fear conditioning is observed [30]. On the other hand, cardiac involvement is less common and survival is better among patients with anti-SRP [31]. Srp54 is also upregulated after contextual fear conditioning in the rat [32]. Furthermore, SRP is related to NMD [33]. SRP and NMD are associated with abnormal gene expression, e.g., miss-splicing, which is suggested to induce PTSD-mediated heart diseases.

Next, to identify what governed the processes overall, 457 gene symbols were uploaded to Enrichr [25] because 457 genes are too many to be considered primary factors of PTSD-mediated heart diseases; a smaller number of factors is preferable. As a result, many genes listed among “Transcription factors PPI” were found to be enriched (41 genes have the adjusted *P*-values less than 0.05; see Additional file 7 for the full list). As for the top 10 proteins (Table 5), many are related to heart diseases and fear memories as shown below. Mutation of the most significant gene, *ILF3*, is related to heart attacks [34]. *ESR1*, the second most significant gene, was reported to be related to fear conditioning [35] and heart diseases [36]. *RAD21*, the third most significant gene, is related to memory formation [37] (through genomic structure of BDNF and Arc) as well as to heart diseases [38]. *HTT*, the fourth most significant gene, is related to heart diseases [39] and its full name is Huntingtin, which is naturally related to the corresponding neurodegenerative disease. *ATF2*, the seventh most significant gene, was reported to be possibly involved in Alzheimer’s and heart diseases [40].

**Table 5:** Top 10 proteins interacting with the 457 genes identified by TD-based unsupervised FE, which were listed by Enrichr (“Transcription factors PPI”)

Term	Overlap	*P*-value	Adjusted *P*-value
ILF3	50/297	6.90E-29	1.59E-26
ESR1	81/871	9.85E-28	1.13E-25
RAD21	37/237	1.89E-20	1.45E-18
HTT	38/293	3.98E-18	2.29E-16
ILF2	28/184	1.81E-15	8.34E-14
ESR2	34/365	4.19E-12	1.61E-10
ATF2	26/237	4.13E-11	1.36E-09
NR3C1	18/239	1.05E-05	3.02E-04
TP53	31/628	5.38E-05	1.12E-03
TP63	12/120	1.99E-05	5.08E-04

ATF2 and c-Jun are parts of AP-1, which is known to be related to fear extinction [41] as well as extinction of contextual fear memory [42]. *NR3C1*, the eighth most significant gene, has frequently been reported to be related to fear emmory [43], and its mutation is reported to be related to muscle strength [44]. GR, encoded by *NR3C1*, is required for fetal heart maturation [45]. *NR3C1* was also reported to be one of the driver genes of PTSD [2] in a study involving a search for the genes causing PTSD-mediated heart diseases. The ninth most significant gene, *TP53*, is one of upstream regulators of pathways associated with the onset of memory deficits in mice [46], although nothing was reported to the tenth significant *TP63*.

**Fig. 5:**
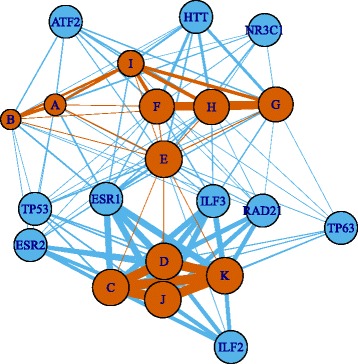
Graphical representation of relations between the identified biological terms and proteins. Biological temrs (orange) and various protein-, DNA-, or mRNA-binding proteins (cyan). A: “heart contraction” (GO BP), B: “cardiac muscle contraction” (GO BP), C: “protein targeted to ER” (GO BP), D: “SRP-dependent cotranslational protein targeted to membrane” (GO BP), E: “neuron part” (GO CC), F: “Huntington’s disease” (KEGG), G: “Parkinson’s disease” (KEGG), H: “Alzheimer’s disease” (KEGG), I: “Cardiac muscle contraction” (KEGG), J: “nonsense-mediated decay (NMD)” (REACTOME), K: “SRP-dependent cotranslational protein targeted to membrane” (REACTOME). Orange edges: genes shared by biological terms, blue edges: genes targeted by protein-, DNA-, or mRNA-binding proteins. Width of edges is proportional to the number of genes. Sizes of the orange circles representing biological terms are proportional to the number of genes enriched in each biological term among the 457 genes

Figure 5 shows the graphical representation of enriched biological terms as well as the 10 above-mentioned interacting proteins. The figure indicates that they are tightly inter-related. Thus, the 10 identified proteins are likely to regulate expression of genes enriched in these biological terms and PTSD-mediated heart diseases as well although additional experimental validation is needed.

To confirm correctness of identification of the enriched biological terms, the 457 gene symbols were also uploaded to MSigDB [47] (Additional file 8). As a result, “Nonsense-mediated decay enhanced by the exon junction complex,” “SRP-dependent cotranslational protein targeted to membrane” (REACTOME), “Parkinson’s disease,” “Alzheimer’s disease,” “Huntington’s disease,” and “Cardiac muscle contraction” (KEGG) were found to be significantly enriched. Therefore, the identified enrichment of these biological terms is trustworthy.

Finally, to confirm the reliability of the selected genes, DrugMatrix in Enricher was analyzed. Many compounds that affect gene expression in the rodent heart were identified; in total, 7,098 combinations of drugs with various dose densities and solvents were found to have the adjusted *P*-values less than 0.01 (Additional file 9). First of all, all the top 10 combinations (Table 6) were found to decrease expression of some genes in the heart, in agreement with the expectation that the identified genes should be related to the heart because they are supposed to contribute to heart failure. In addition, many adverse effects caused by these drugs, as shown in Table 6, are also associdated with PTSD-mediated heart diseases. For example, the most significant drug, low-dose prednisolone [48], increases long-term risk of ischemic cerebrovascular events. Long-term administration of the second most significant drug, ethosuximide, adversely affects fear memory [49]. The third most significant compound, caffeine, is known to be related to heart diseases [50] as well as acquisition and retention of Pavlovian conditioned freezing [51]. The fourth most significant drug, clomipramine, was once suggested to be used for the treatment of anxiety [52]. The fifth most significant drug, prednisolone, was reported to alleviate adverse cardiac effects [53]. Several cases of cardiac adverse reactions related to the seventh and ninth most significant drug, vinorelbine, have been reported in the literature [54]. The eighth most significant drug, atropine, affects heart rate [55]. Cardiac arrest was reported after administration of the tenth most significant drug, oxymetazoline (nasal spray) [56]. These relations between heart problems or fear memory and drugs downregulating expression of selected genes in the heart support the reliability of our findings, too.

**Table 6:** Top 10 significant drugs identified by DrugMatrix in Enricher

Candidate drugs	Overlap	*P*-value	Adjusted *P*-value
Prednisolone-184_mg/kg_in_Water-Rat-Heart-5d-dn	53/343	1.10E-28	4.34E-25
Ethosuximide-1200_mg/kg_in_Water-Rat-Heart-3d-dn	50/319	2.16E-27	5.66E-24
Caffeine-93_mg/kg_in_Water-Rat-Heart-3d-dn	51/345	1.07E-26	1.69E-23
Clomipramine-115_mg/kg_in_Water-Rat-Heart-3d-dn	49/320	2.19E-26	2.55E-23
Prednisolone-184_mg/kg_in_Water-Rat-Heart-3d-dn	53/340	7.07E-29	4.34E-25
Gatifloxacin-770_mg/kg_in_Corn_Oil-Rat-Heart-1d-dn	48/315	9.12E-26	7.19E-23
Vinorelbine-1.5_mg/kg_in_Saline-Rat-Heart-1d-dn	51/351	2.45E-26	2.55E-23
Atropine-94_mg/kg_in_Water-Rat-Heart-5d-dn	51/353	3.22E-26	2.82E-23
Vinorelbine-1.5_mg/kg_in_Saline-Rat-Heart-3d-dn	47/305	1.83E-25	1.31E-22
Oxymetazoline-0.5_mg/kg_in_Water-Rat-Heart-5d-dn	48/325	3.77E-25	2.47E-22

Drug names, concentrations, solvents, period after treatment, and up- or downregulation (dn) are listed. Overlap means the number of genes among the 457 genes identified by TD-based unsupervised FE

Before closing this subsection, I would like to comment on some points. First, comparisons with some related works. Since Vaccarino *et al* [4] has clearly denoted that there are limited number of mutated genes shared between PTSD and congenital heart defect (CHD), it might not look reasonable that I argued that genomic background was important. However, even if there are no shared mutated genes between PTSD and CHD, genomic background can induce association between PTSD and CHD. For examples, there are two genes A and B. A is a CHD causing genes and B is regulating A. Then, even if mutation of B takes place not in CHD but in PTSD, genomic background (i.e., mutation of gene B) still can induce CHD. This means that shared mutated genes is not only genomic background that can induce the association between PTSD and CHD. Cho *et al* [57] also investigated mRNA and miRNA expression of stressed mouse heart. Nevertheless, since we have extensively studied this study in our previous study [7], I did not discuss about it in the present paper. Finally, Pollard *et al* [2] identified 37 mutated genes shared between PTSD and cardiovascular disease (CVD). However, as in the case of PTSD and CHD, shared mutated genes are not only factors that can mediate PTSD mediated heart disease. Actually, there are no significant overlaps between these 37 genes and our 457 genes. Possibly, our identified gene expression alteration between PTSD and controls is not due to a direct effect of shared mutated genes but due to more complicated indirect effect. Second, I would like to briefly argue about how TD can figure out hidden relations among genes and diseases. From the mathematical point of views, TD is nothing but possible assumption. Thus, the validation of methodology can be done only based on the goodness of outcomes. Since our results are biologically reliable, our assumption that gene expression has hidden structure that can be figured out TD seems to be correct. More applications of this strategy will add more confidence to TD based unsupervised FE in the future.

## Conclusions

In this paper, TD-based unsupervised FE was applied to murine tissue gene expression profiles with and without stress conditions. The resulting 457 genes associated with 801 probes identified as outliers using gene singular value vectors were subjected to various enrichment analyses. Ten proteins likely to regulate expression of these genes are proposed here as possible causal genes of PTSD-mediated heart diseases.

## Additional files

Additional file 1 Mean expression profiles of 10 clusters in synthetic data. Mean gene expression profiles of the 10 clusters from Table 1 for synthetic data when the Ward method was employed. (PDF 17 kb)

Additional file 2Other tissue singular value vectors. $x_{\ell 1_{2} eq 4,j_{2}}$ for gene expression profiles. (PDF 10 kb)

Additional file 3 801 probe IDs. Probe IDs identified by TD-based unsupervised FE. (CSV 11 kb)

Additional file 4 Comparison with other methods. A supporting document about the comparisons with other methods. (PDF 948 kb)

Additional file 5 457 gene symbols. Gene symbols associated with 801 probes. (CSV 3 kb)

Additional file 6 g:profiler. A full list of enrichment analyses of g:profiler. (XLSX 697 kb)

Additional file 7 Transcription factors PPI. A full list of enrichment analyses of transcription factors PPI provided by Enrichr. (CSV 70 kb)

Additional file 8 MSigDB. A full list of enrichment analyses of DrugMatrix provided by MSigDB. (CSV 193 kb)

Additional file 9 DrugMatrix. A full list of enrichment analyses of DrugMatrix provided by Enrichr. (CSV 4 kb)

## Abbreviations

AY: Amygdala
BH: Benjamini–Hochberg
BP: Biological process
CC: Cellular component
FE: Feature extraction
GEO: Gene expression omnibus
GO: Gene Ontology
HC: Hippocampus
HOSVD: Higher-order singular value decomposition
KEGG: Kyoto Encyclopedia of Genes and Genomes
MF: Molecular function
MPFC: Medial prefrontal cortex
NMD: Nonsense-mediated decay
PTSD: Post-traumatic stress disorder
SE: Septal nucleus
SRP: Signal recognition particle
ST: Striatum
TD: Tensor decomposition
VS: Ventral striatum
