# Supplementary figures and images for: Tensor decomposition-based unsupervised feature extraction identifies candidate genes that induce post-traumatic stress disorder-mediated heart diseases
Source: BMC Med Genomics. 2017 Dec 21;10(Suppl 4):67. doi: 10.1186/s12920-017-0302-1 (PMC5763504; doi:10.1186/s12920-017-0302-1)

## cluster 1

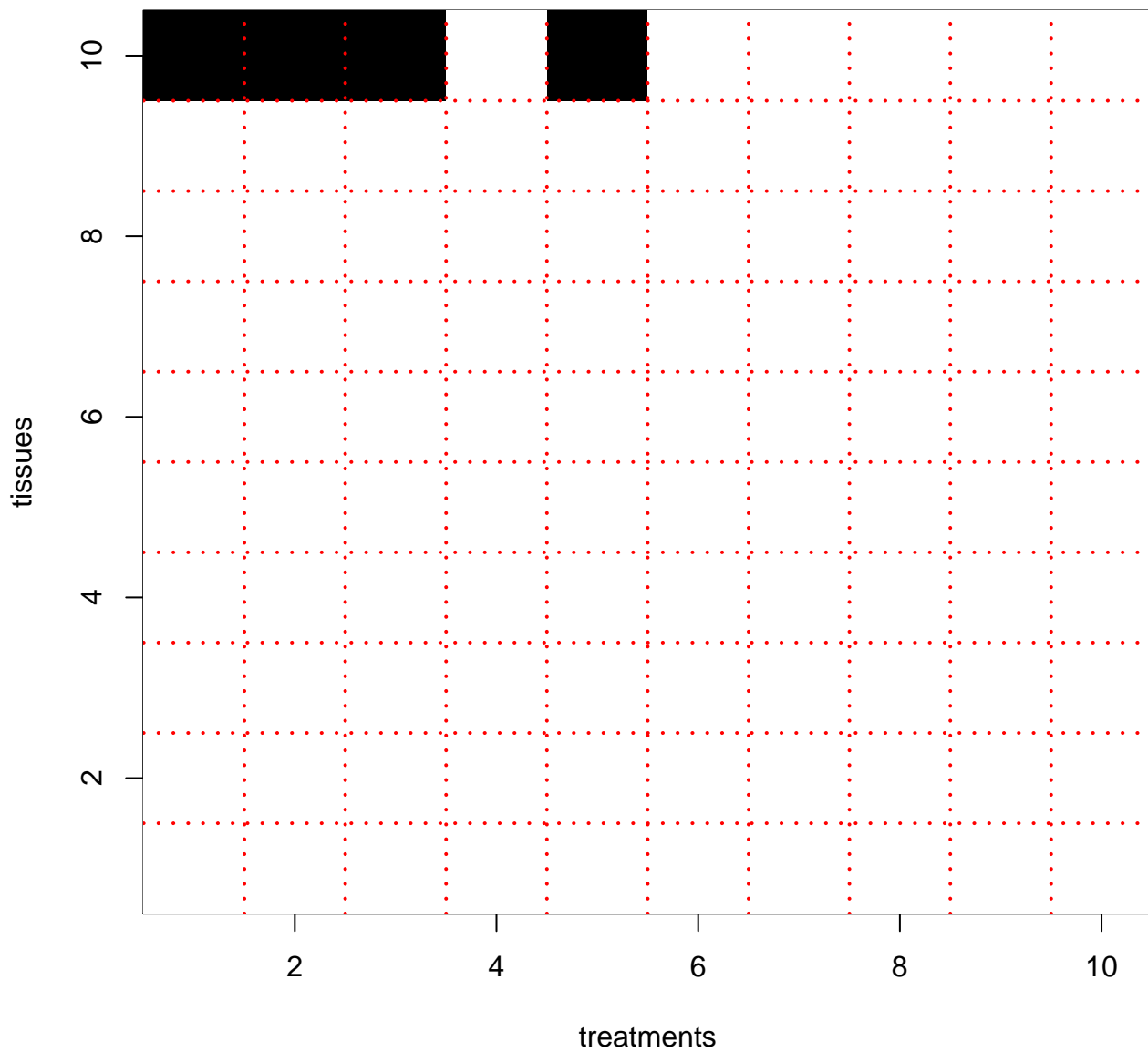

## cluster 2

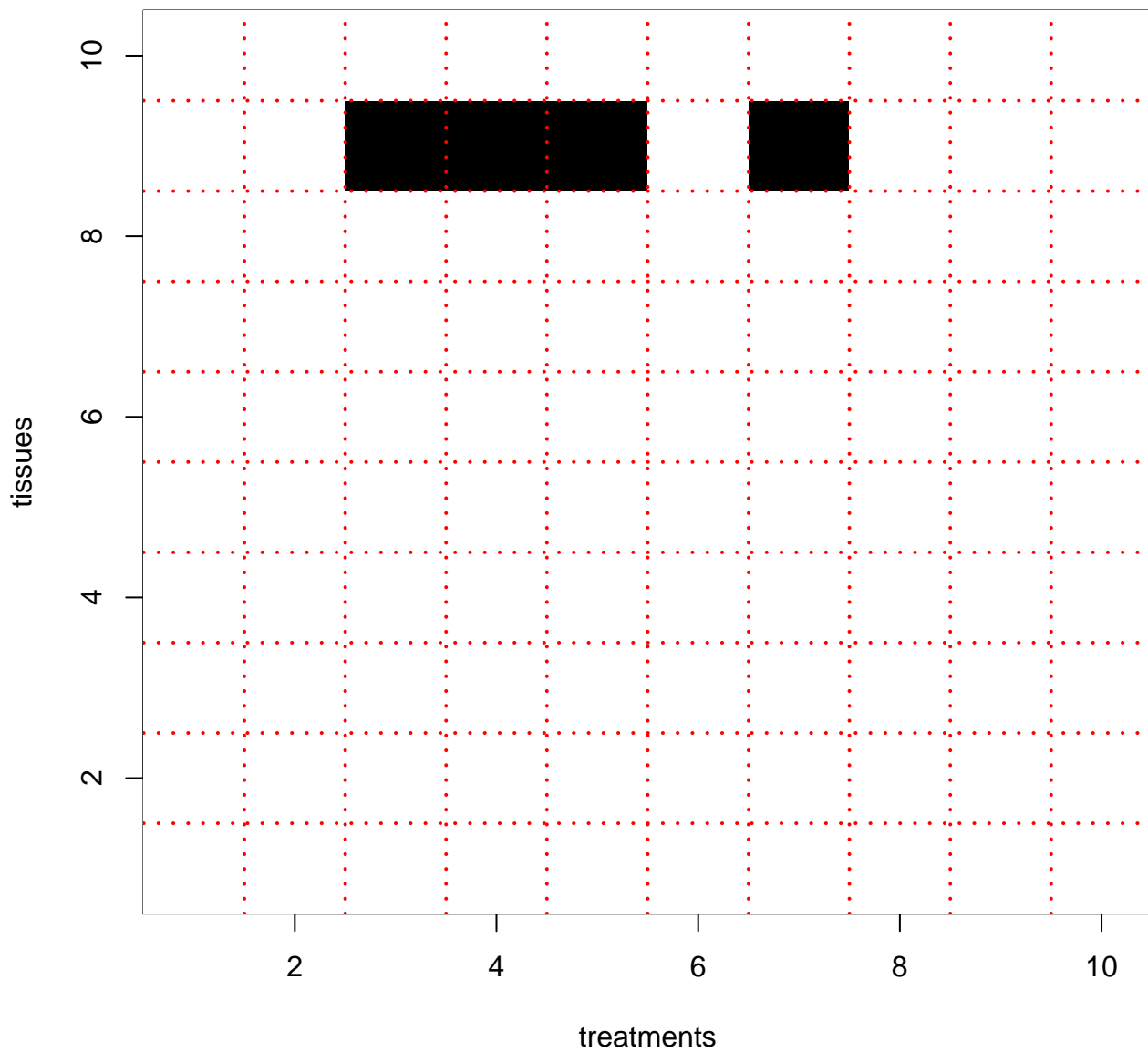

### cluster 3

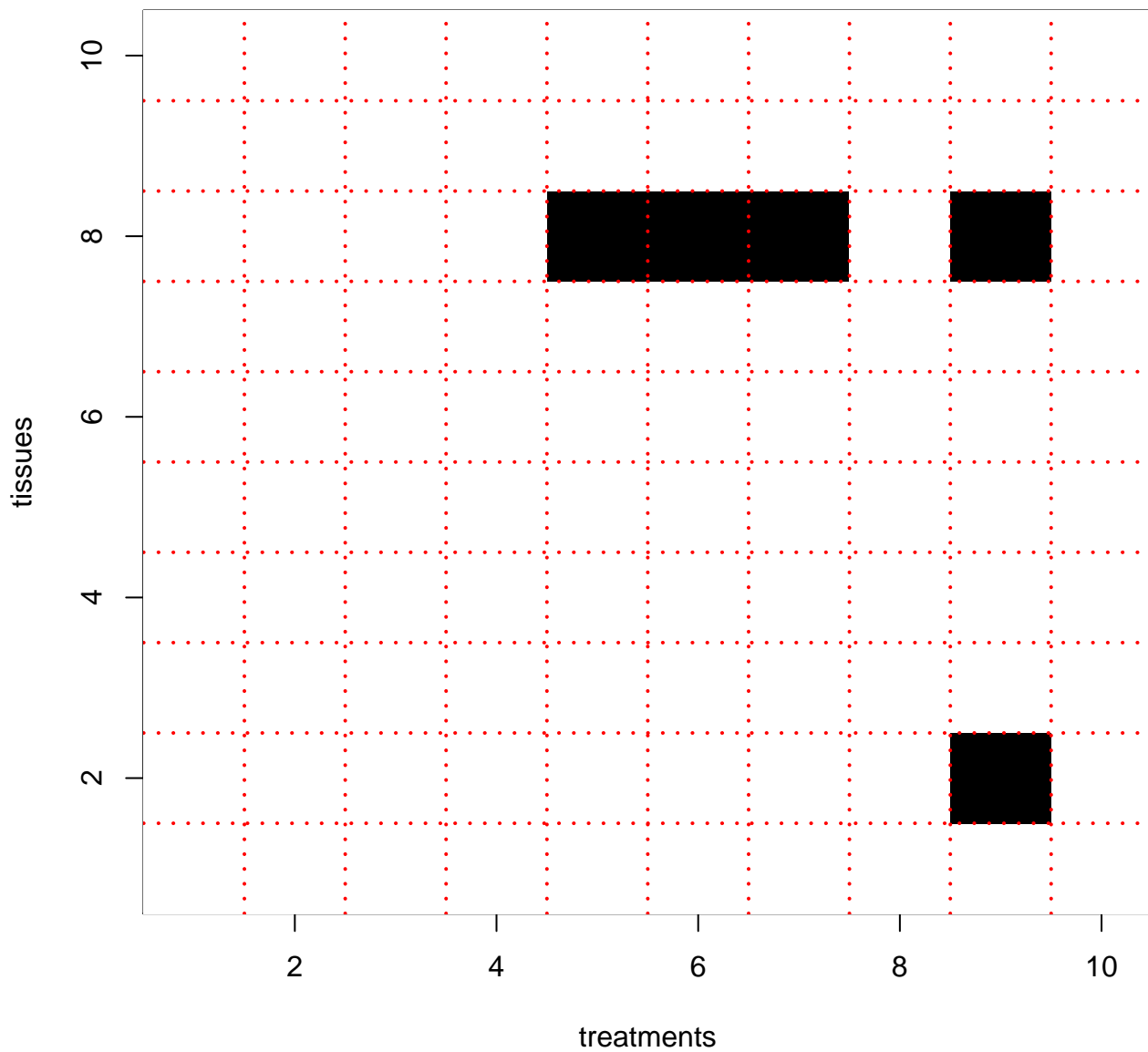

**cluster 4**

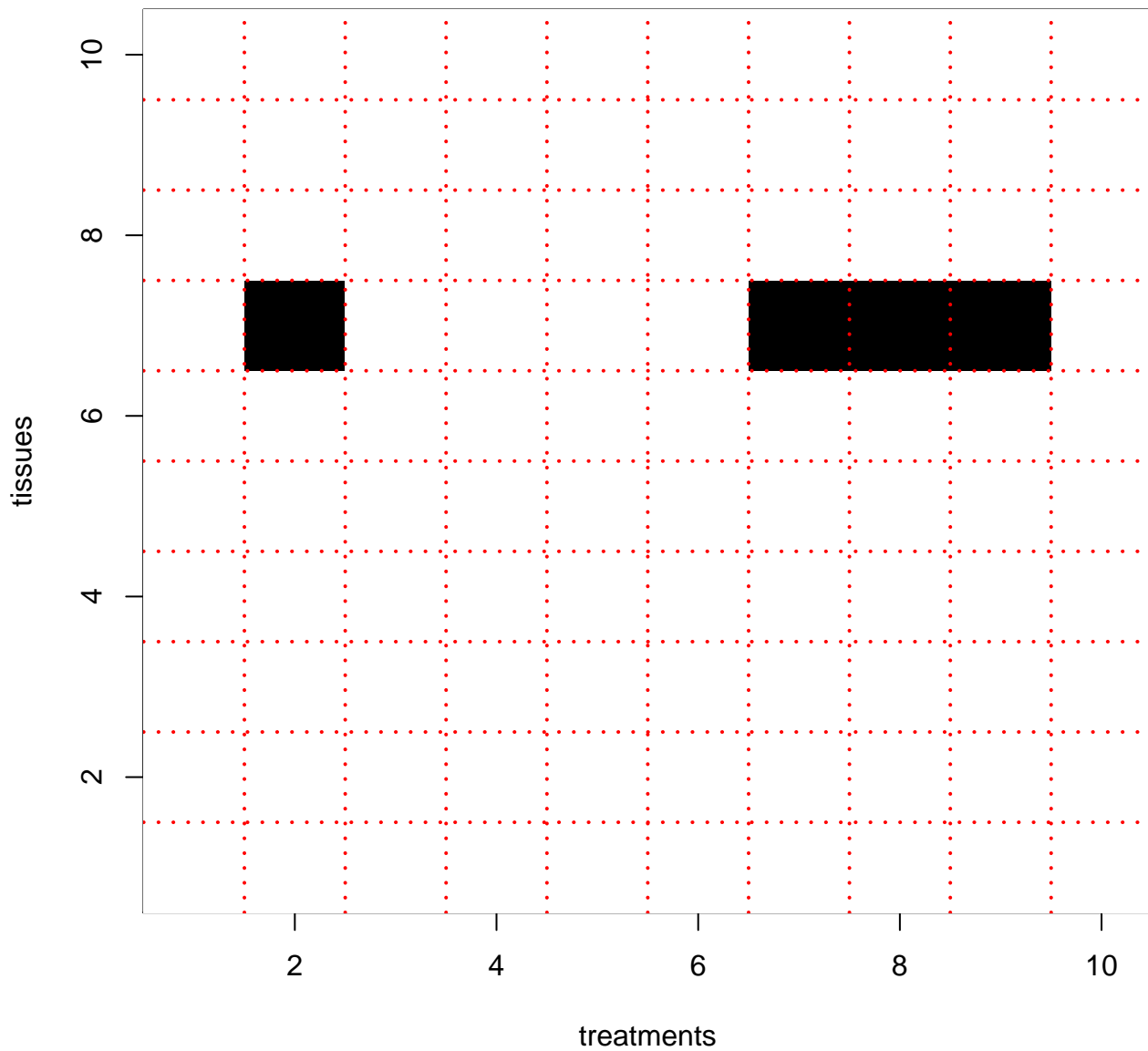

## cluster 5

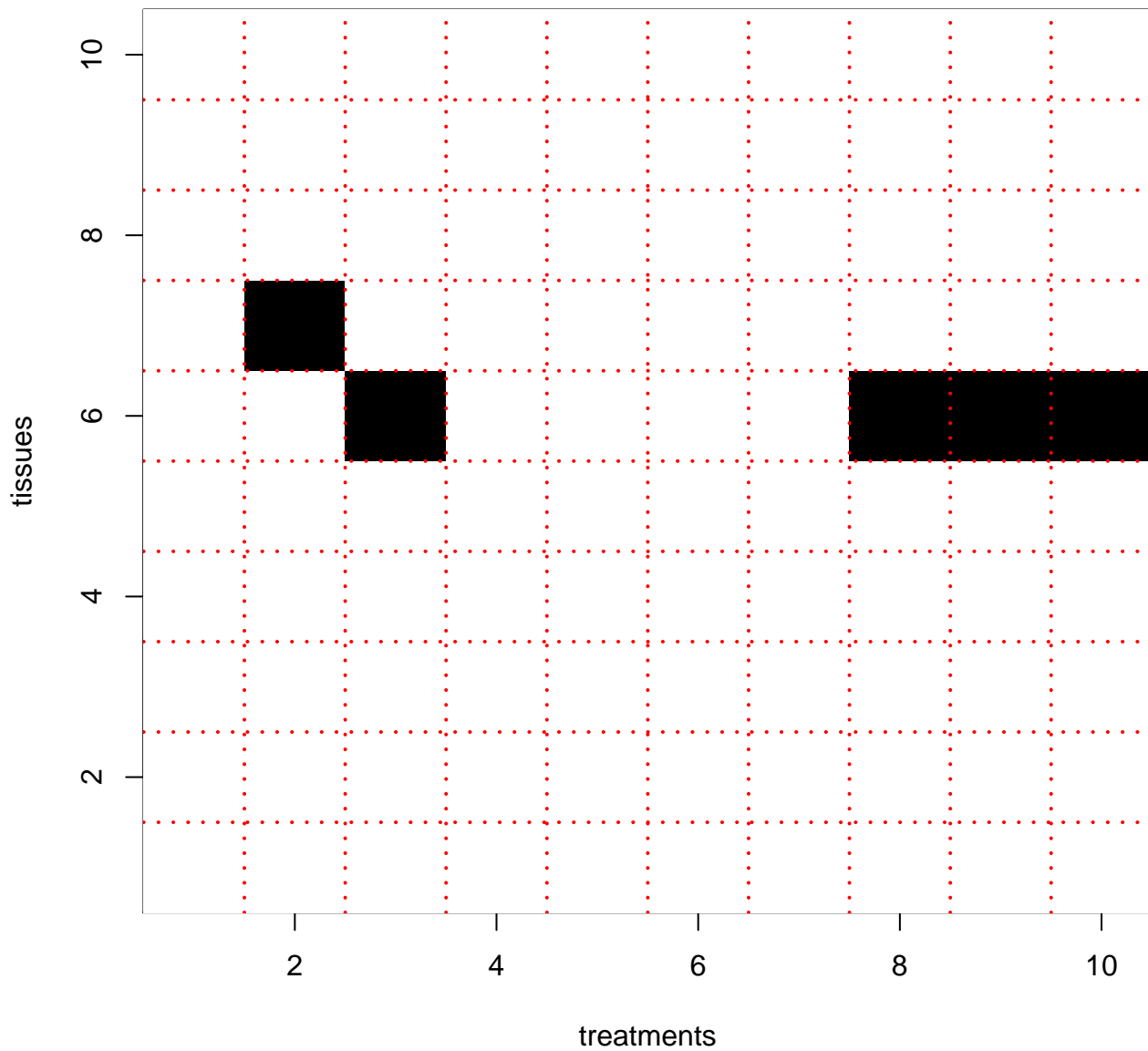

## cluster 6

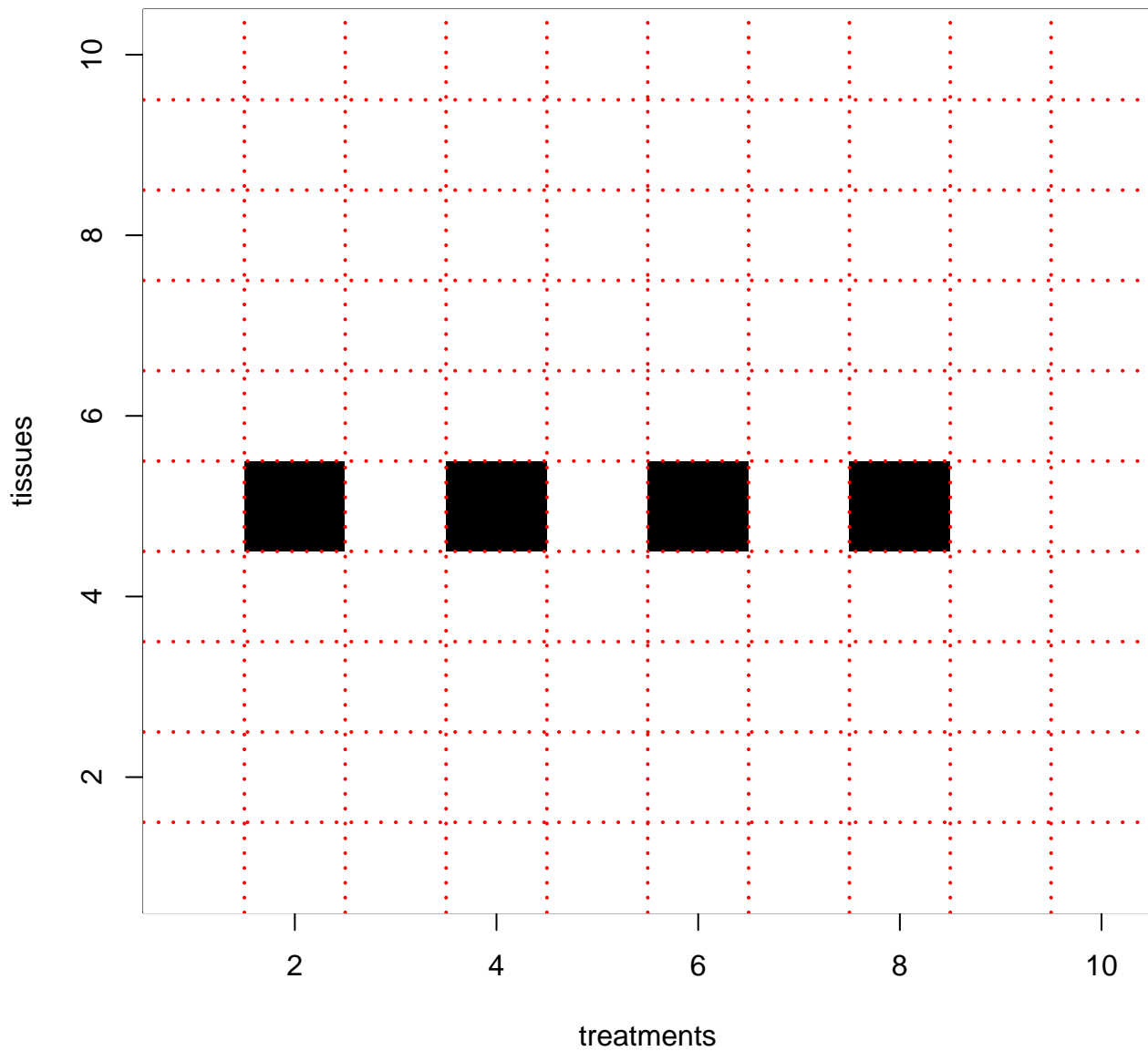

**cluster 7**

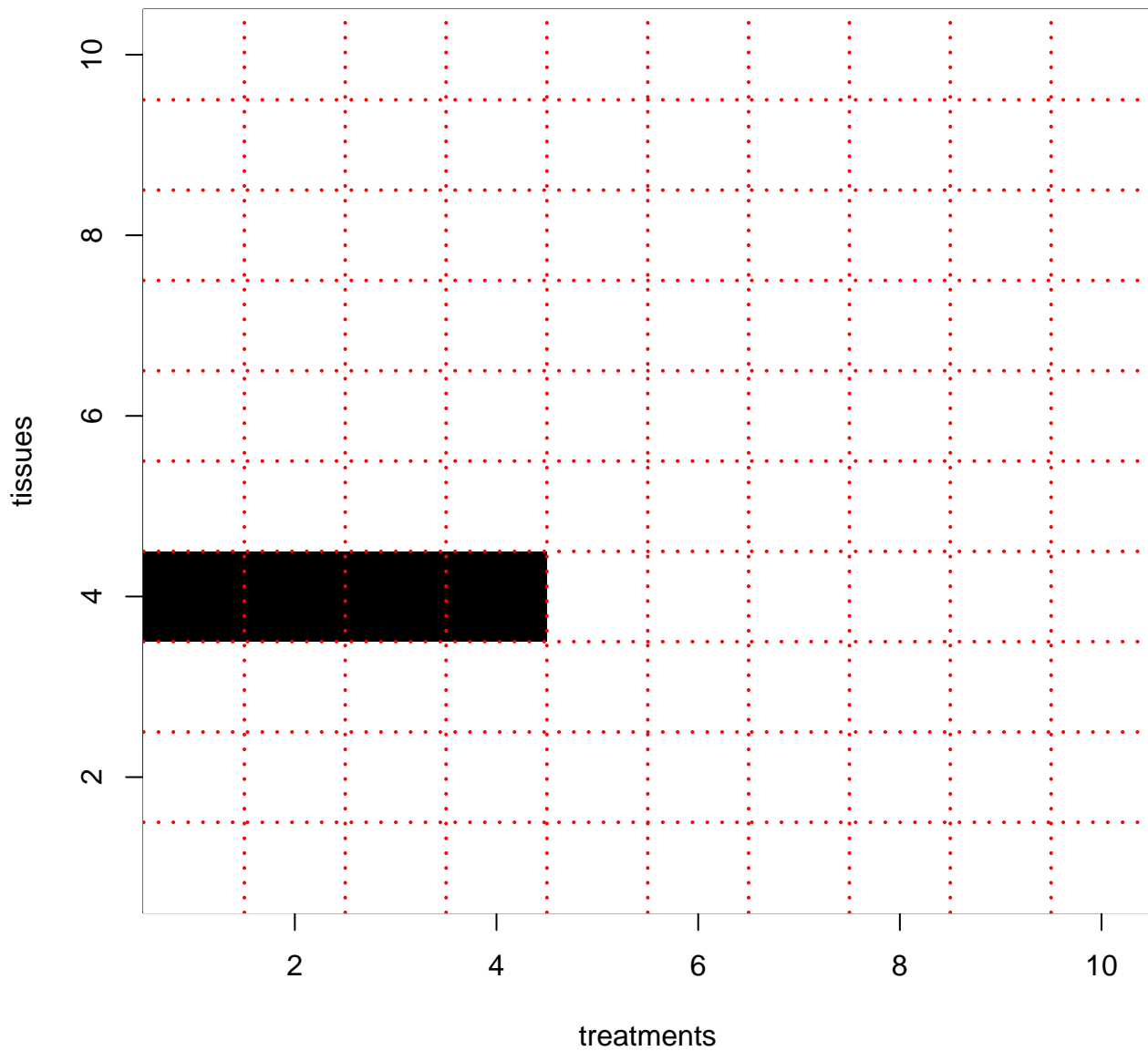

## cluster 8

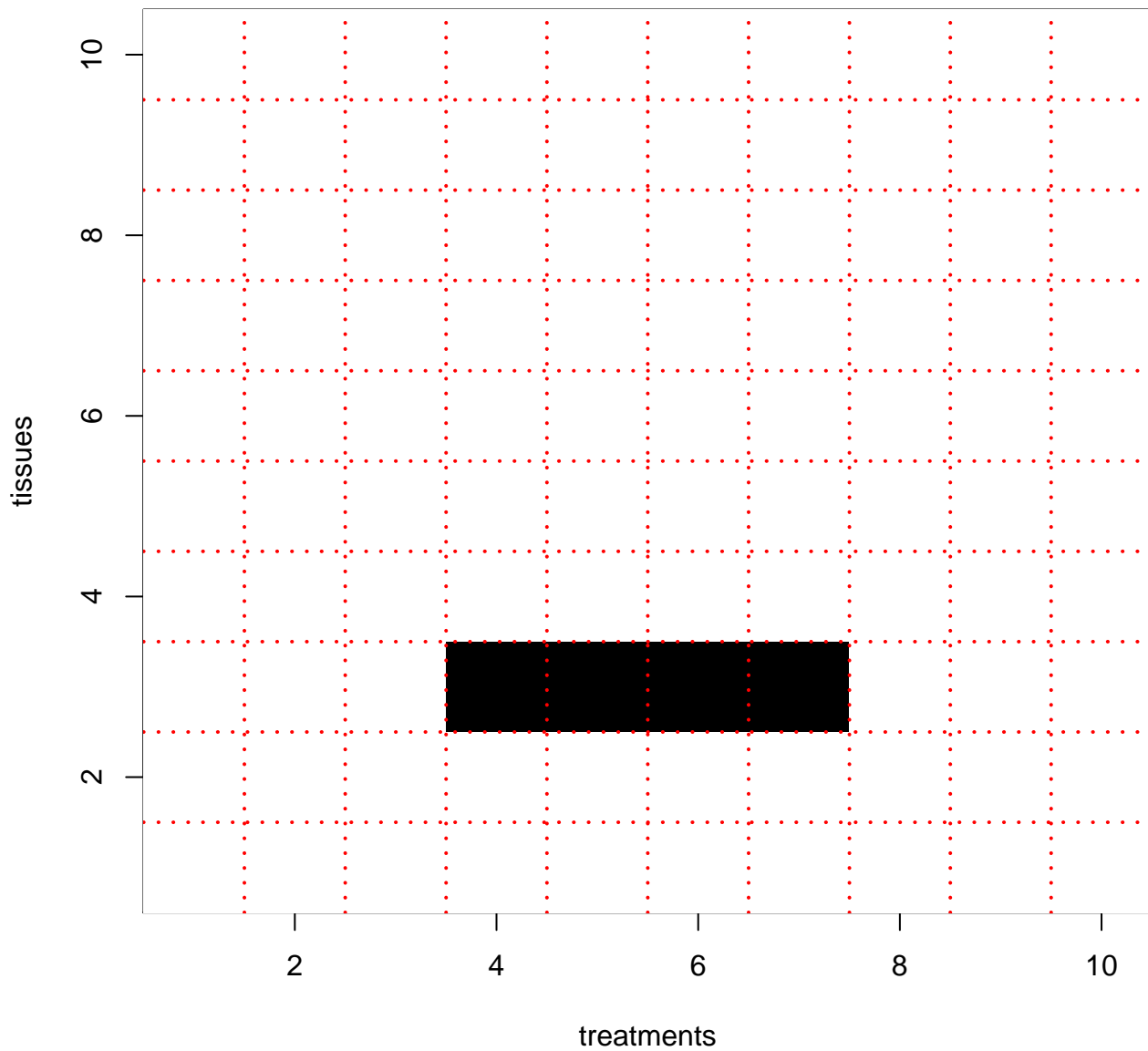

**cluster 9**

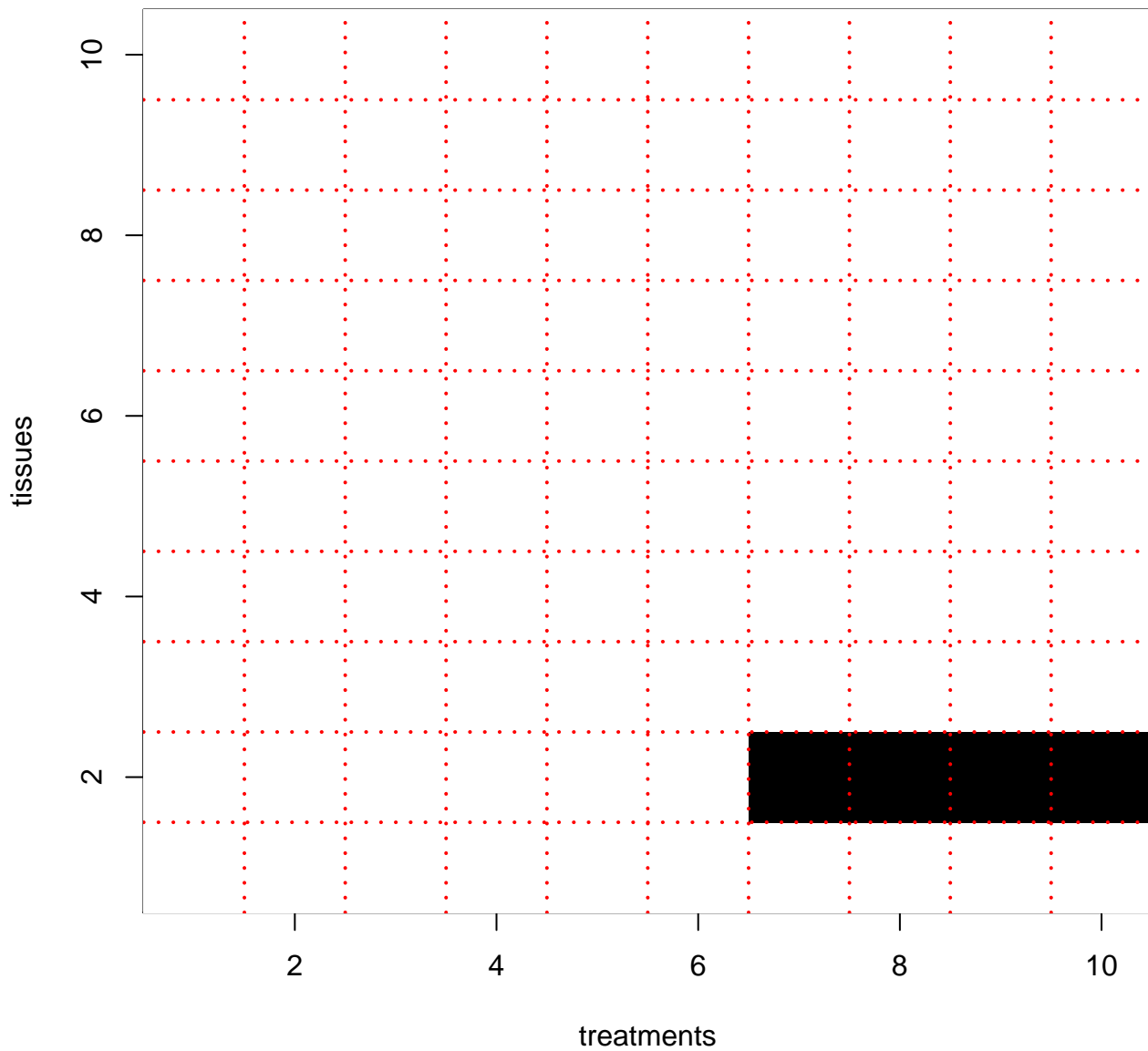

# cluster 10

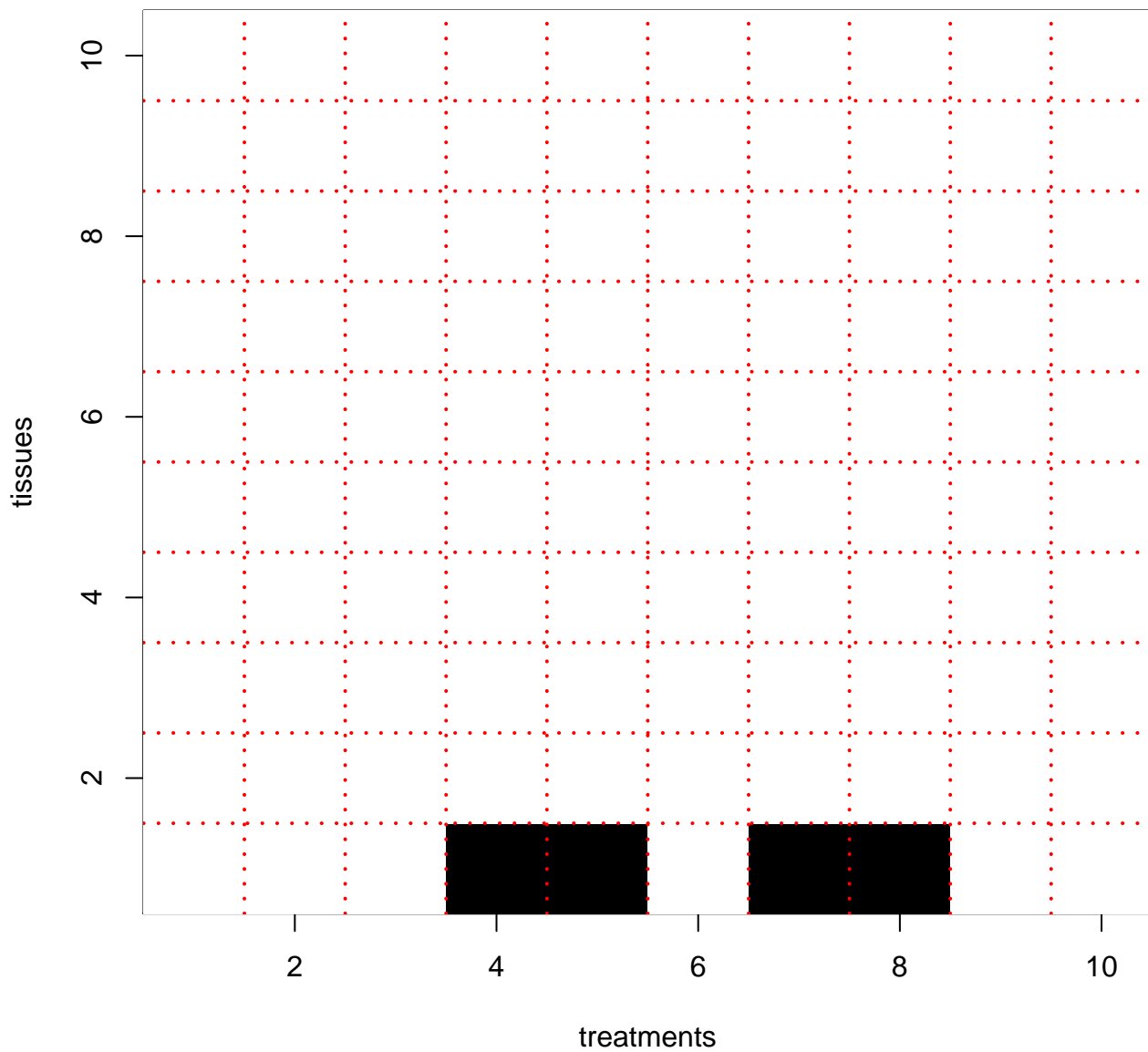

## cluster 11

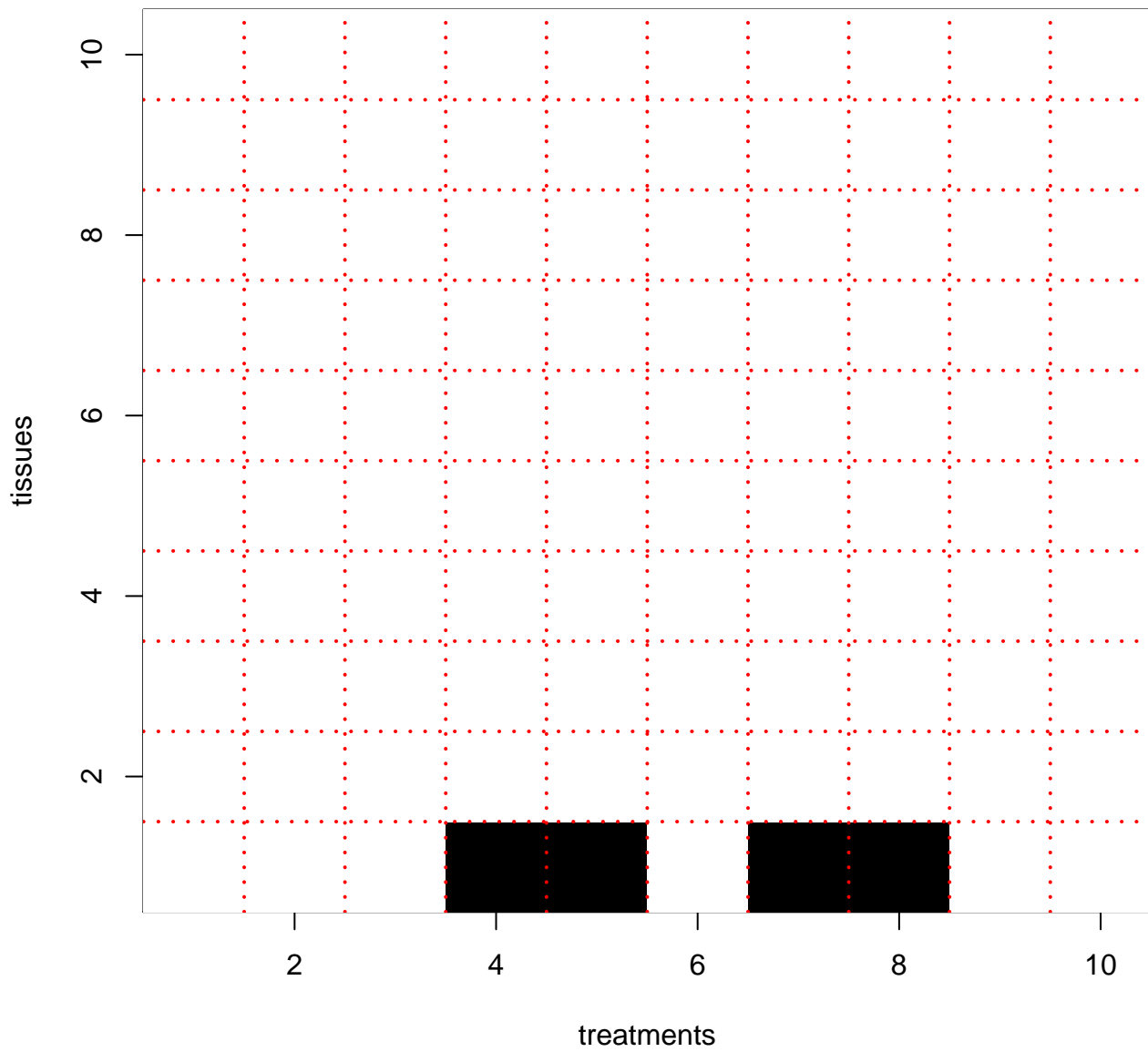

Supplement: Supplementary file 1 — Mean expression profiles of 10 clusters in synthetic data. Mean gene expression profiles of the 10 clusters from Table 1 for synthetic data when the Ward method was employed. (PDF 17 kb) [file 12920_2017_302_MOESM1_ESM.pdf]
