# Supplementary material for: Tensor decomposition-based unsupervised feature extraction identifies candidate genes that induce post-traumatic stress disorder-mediated heart diseases
Source: BMC Med Genomics. 2017 Dec 21;10(Suppl 4):67. doi: 10.1186/s12920-017-0302-1 (PMC5763504; doi:10.1186/s12920-017-0302-1)
Supplement: Supplementary file 2 — Other tissue singular value vectors. $x_{\ell 1_{2} eq 4,j_{2}}$xℓ12≠4,j2 for gene expression profiles. (PDF 10 kb) [file 12920_2017_302_MOESM2_ESM.pdf]

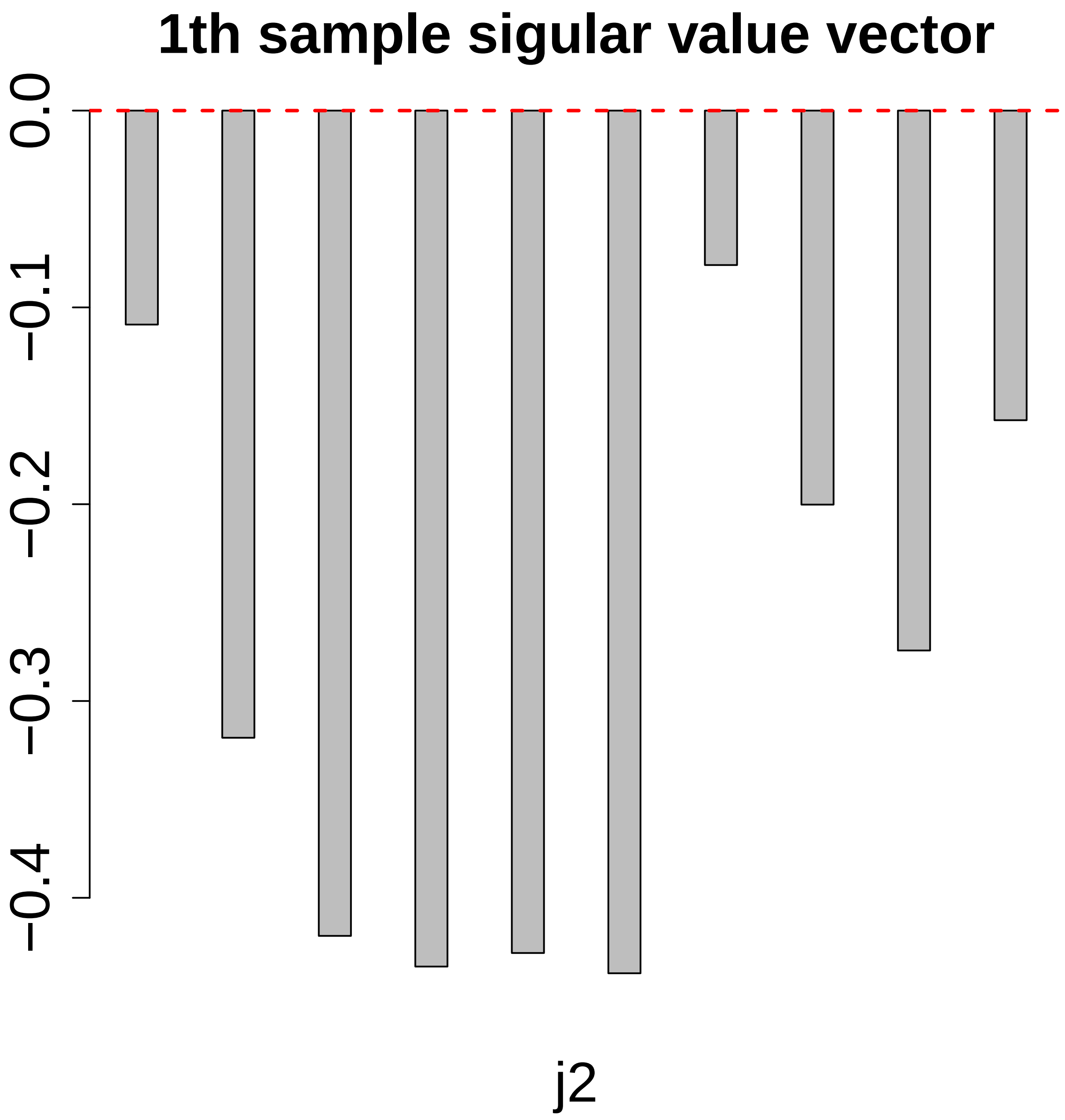
1th sample sigular value vector
0.0
−0.1
−0.2
−0.3
−0.4
j2

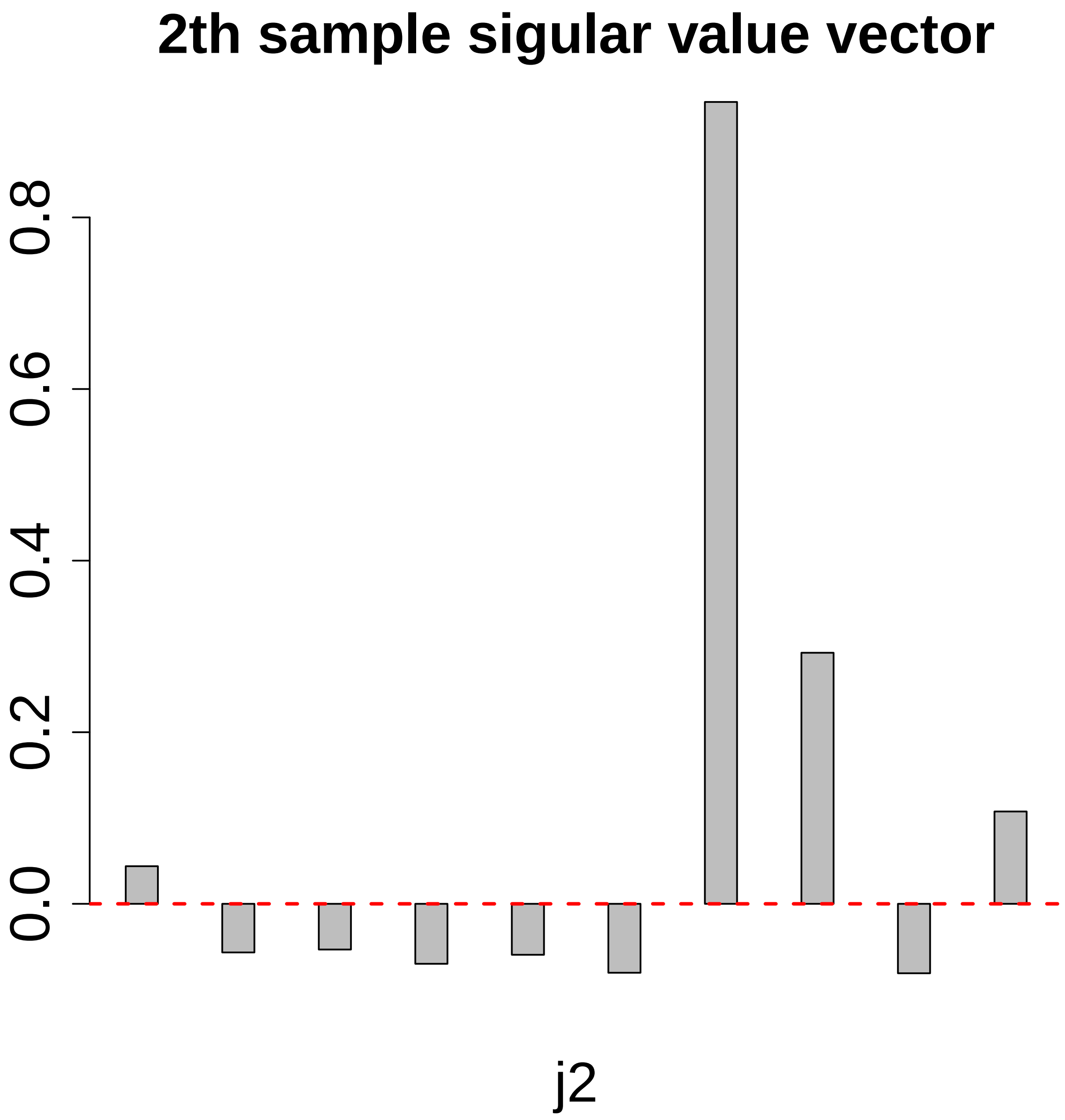
2th sample sigular value vector
0.0
0.2
0.4
0.6
0.8
j2

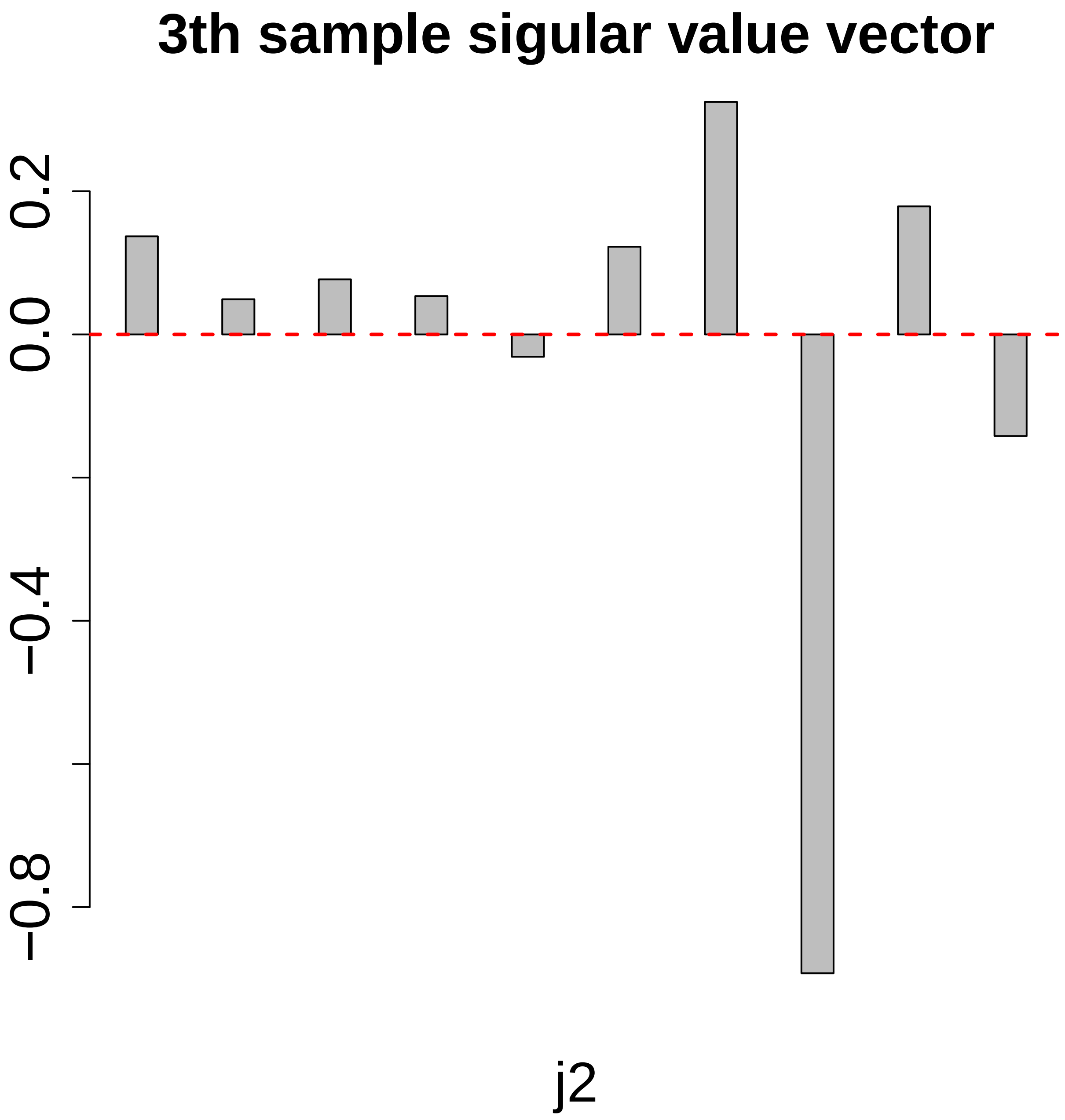
3th sample sigular value vector
0.2
0.0
−0.4
−0.8
j2

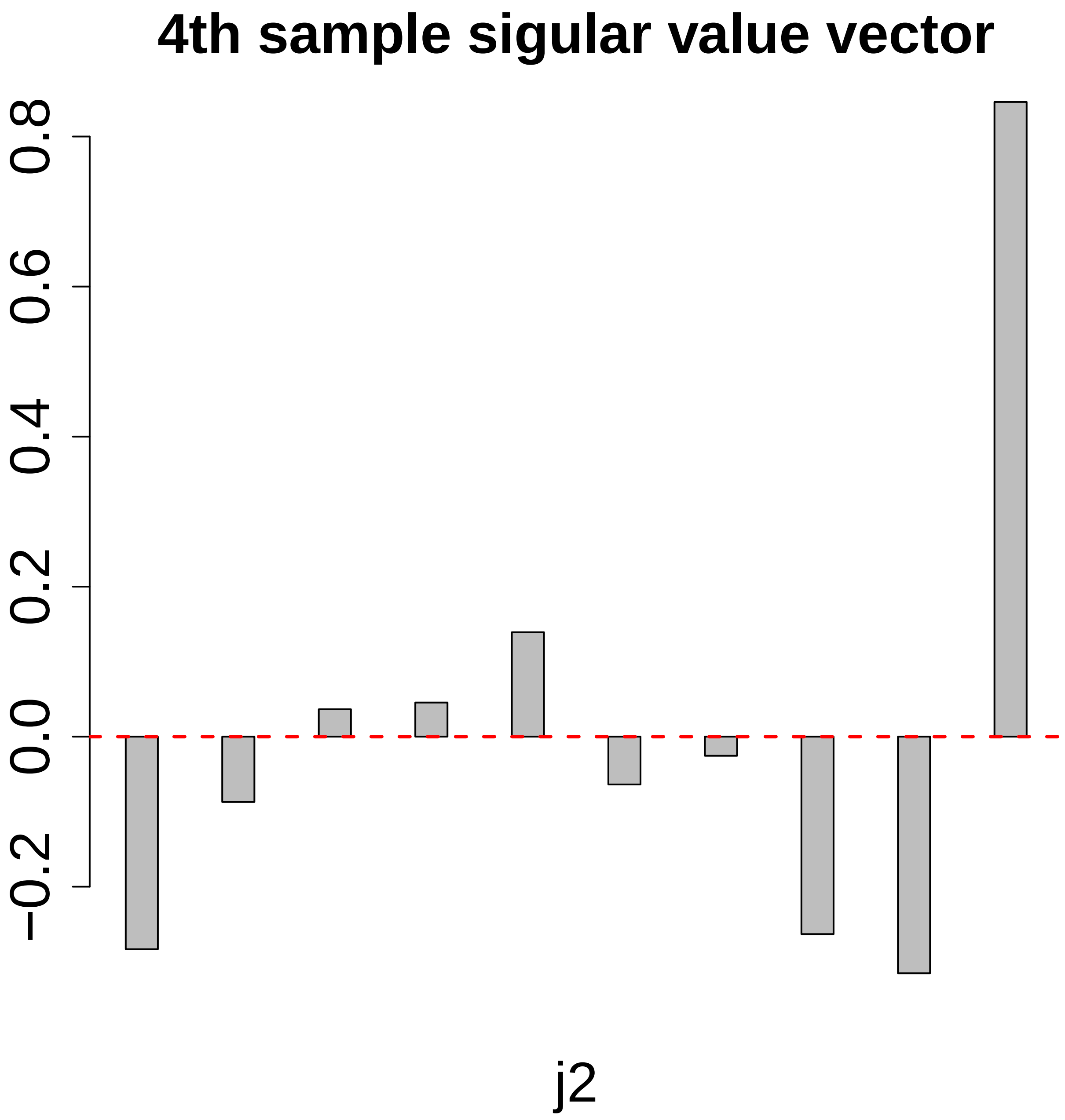
4th sample sigular value vector
0.8
0.6
0.4
0.2
0.0
−0.2
j2

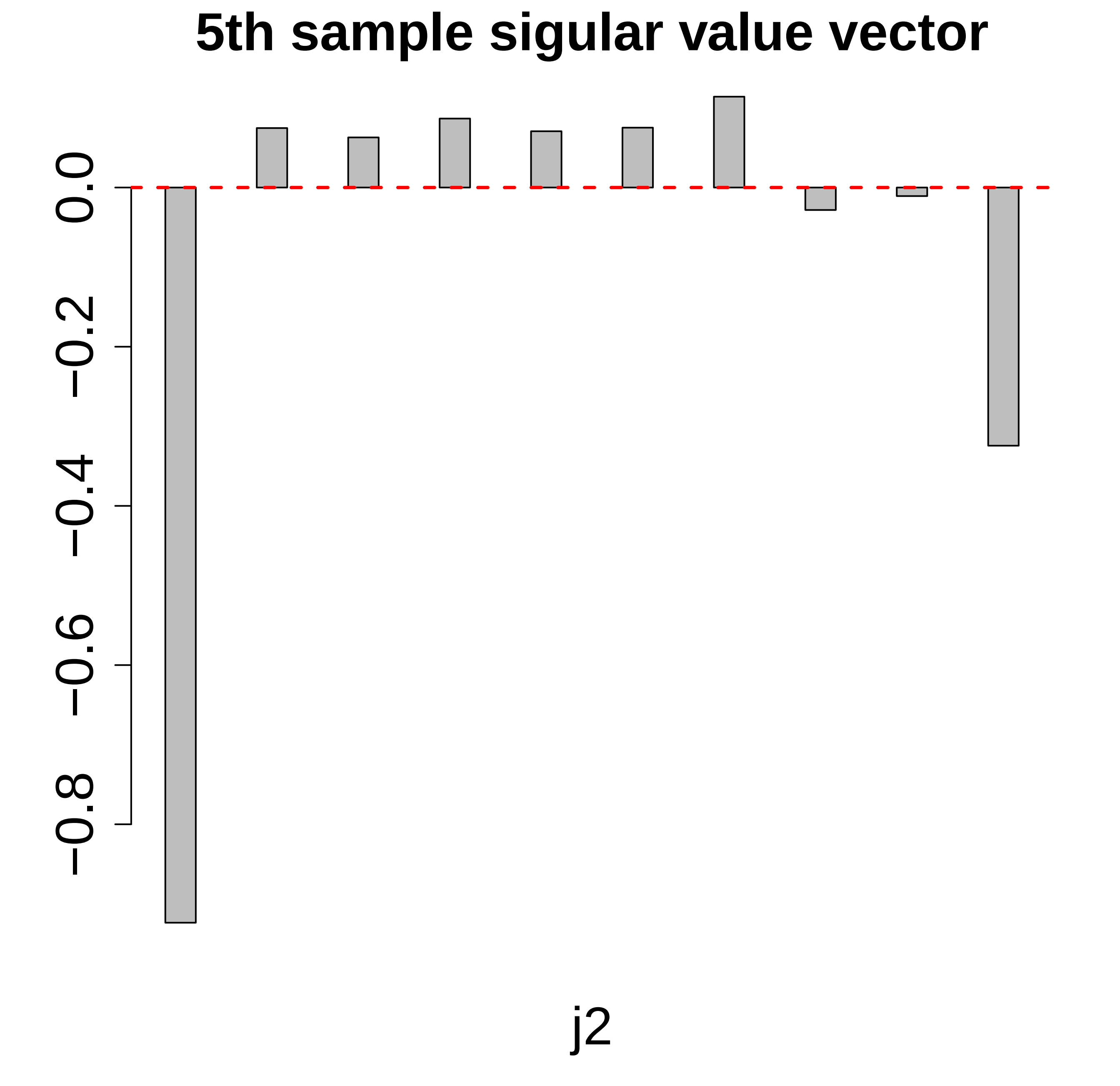
5th sample sigular value vector
0.0
−0.2
−0.4
−0.6
−0.8
j2

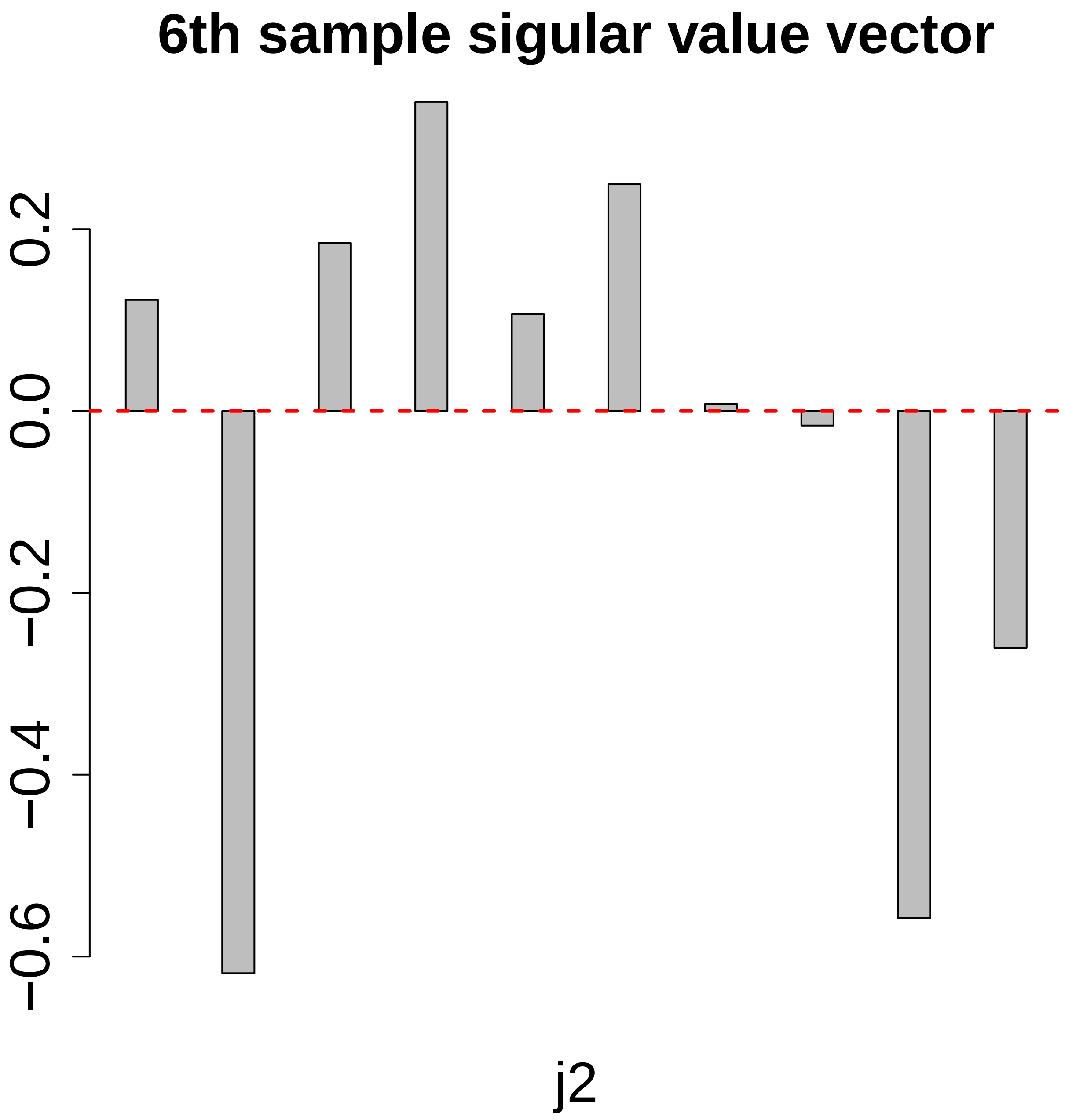
6th sample sigular value vector
0.2
0.0
−0.2
−0.4
−0.6
j2

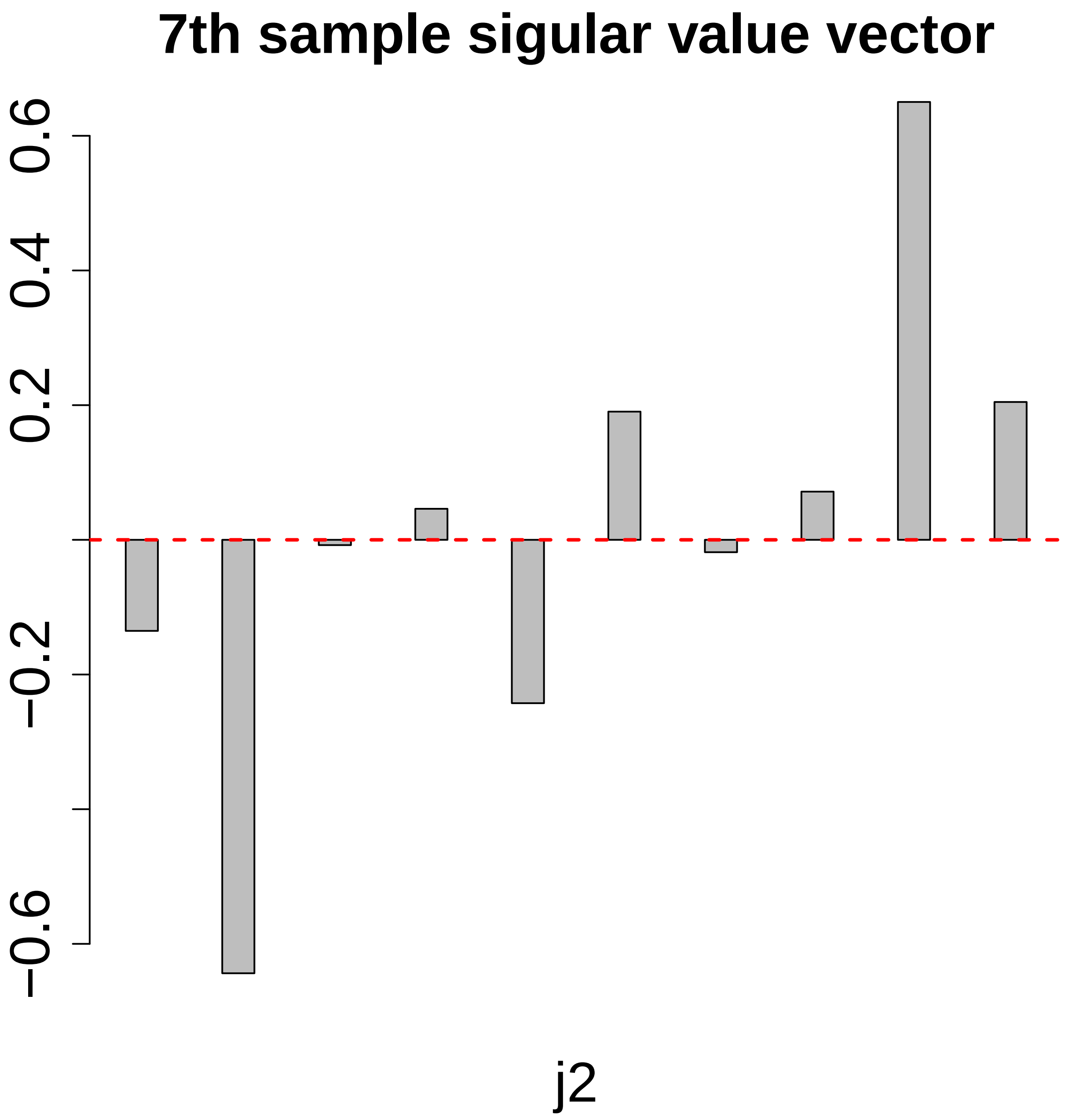
7th sample sigular value vector
0.6
0.4
0.2
−0.2
−0.6
j2

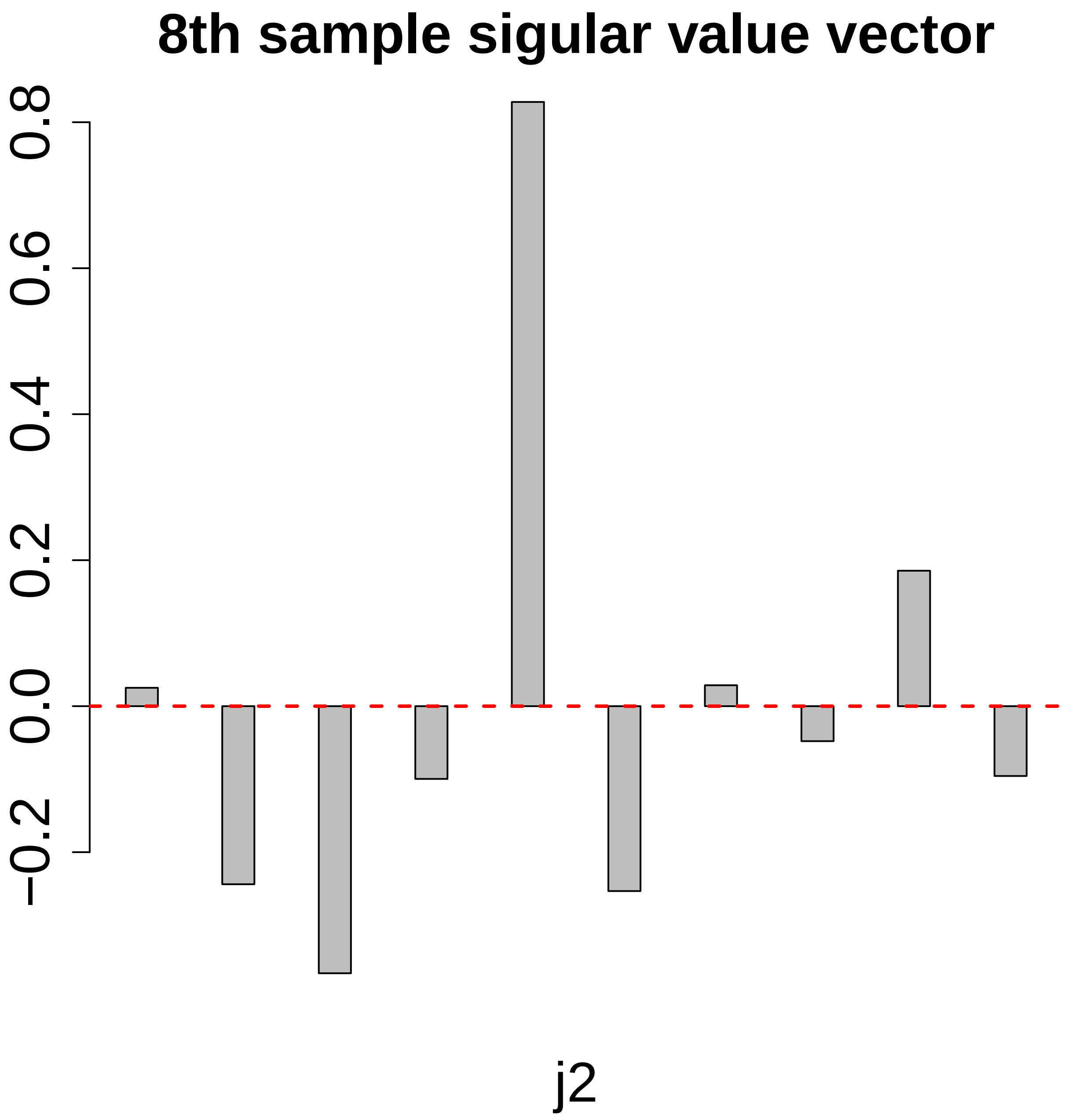
8th sample sigular value vector
0.8
0.6
0.4
0.2
0.0
−0.2
j2

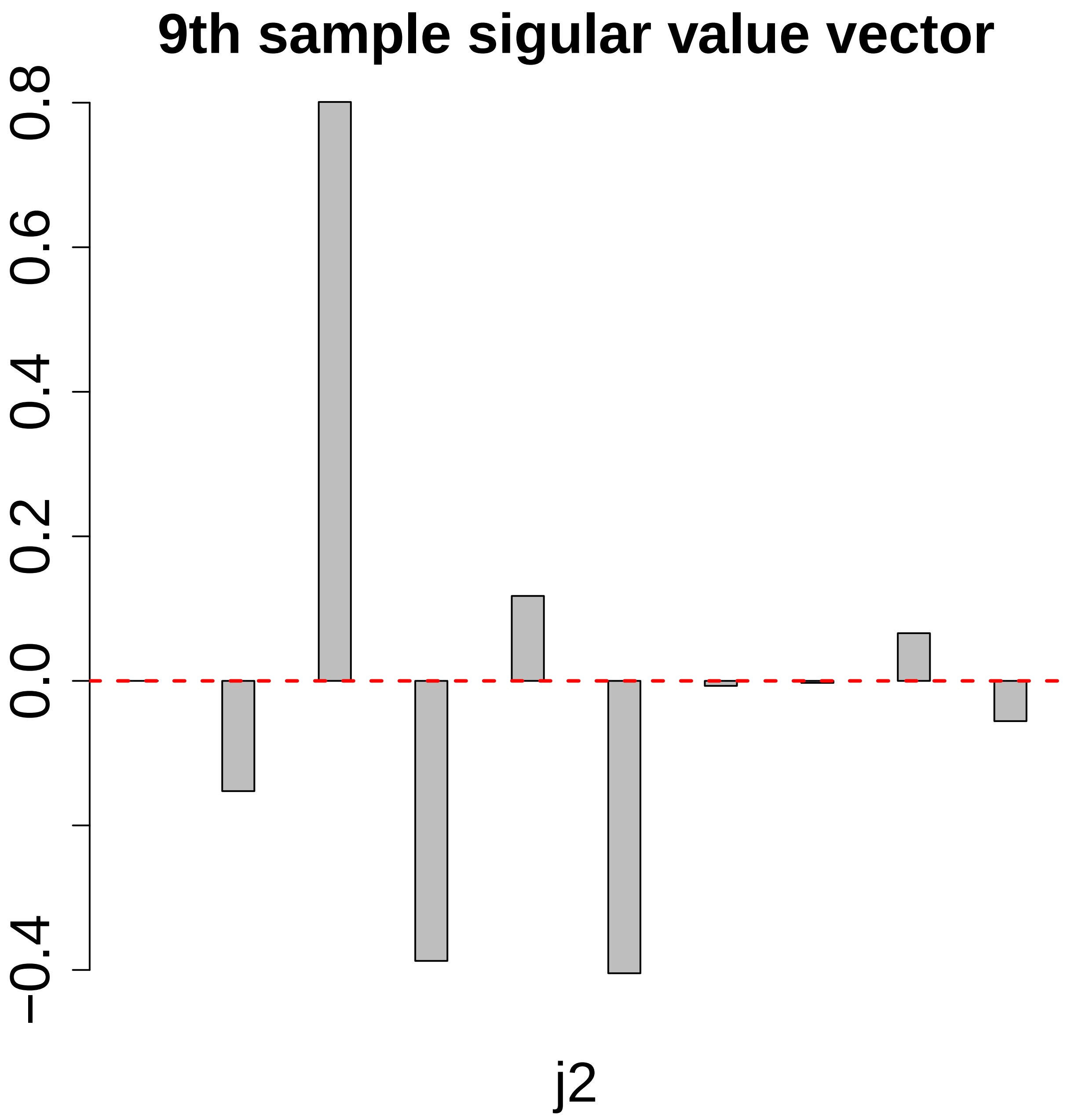
9th sample sigular value vector
0.8
0.6
0.4
0.2
0.0
−0.4
j2

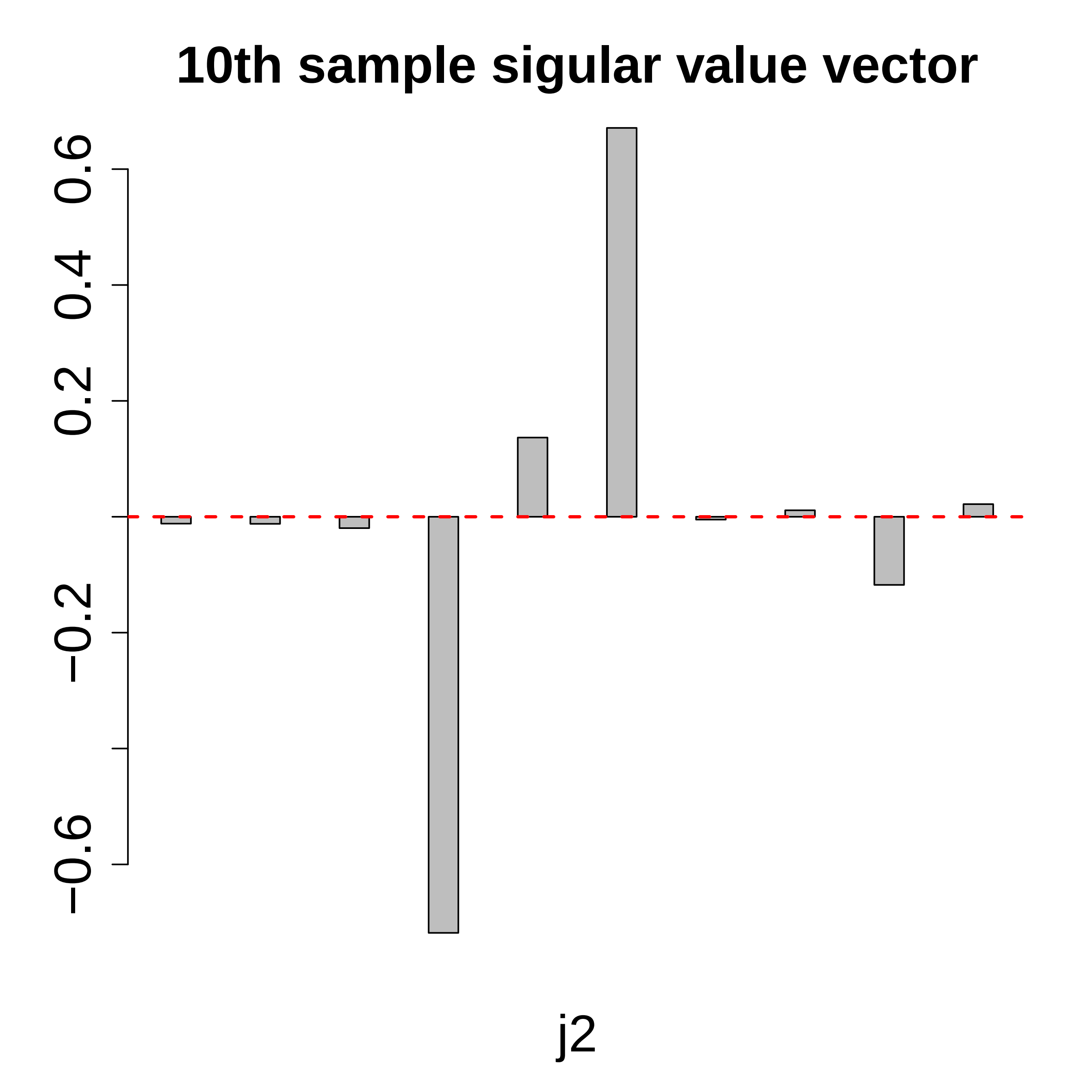

10th sample sigular value vector
0.6
0.4
0.2
−0.2
−0.6
j2
