# Supplementary material for: Tensor decomposition-based unsupervised feature extraction identifies candidate genes that induce post-traumatic stress disorder-mediated heart diseases
Source: BMC Med Genomics. 2017 Dec 21;10(Suppl 4):67. doi: 10.1186/s12920-017-0302-1 (PMC5763504; doi:10.1186/s12920-017-0302-1)
Supplement: Supplementary file 4 — Comparison with other methods. A supporting document about the comparisons with other methods. (PDF 948 kb) [file 12920_2017_302_MOESM4_ESM.pdf]

Supplementary Document: Comparison with other methodologies

Tensor decomposition-based unsupervised feature extraction identifies candidate genes that induce post-traumatic stress disorder mediated heart diseases

Y-h. Taguchi, tag@granular.com, Dept. Phys., Chuo Univ., Tokyo 112-8551, Japan

Abstract

To see if the other methodologies can perform as well as TD-based unsupervised FE, we applied various methodologies to the data sets analysed in this study.

1 Synthetic data

1.1 Categorical regression

Categorical regression (CR), also known as ANOVA, was the first alternative method applied to the synthetic data set. In CR, x_{ij}, which is expression of the ith gene of the jth sample, is assumed to obey

$$x_{ij} = C_i + \sum_s C_{si}\delta_{sj}$$

where summation was taken over all of the classes and $\delta_{sj} = 1$ only when the jth sample belongs to the sth class, or otherwise $\delta_{sj} = 0$. C_i and C_{si} are regression coefficients. Regression analysis was performed using the lm function in R. P-values attributed to the ith gene were also computed by lm and adjusted by the BH criterion.

As discussed in the main text, because there is only one sample per class, regression analysis is impossible to apply to the synthetic data as it is. Thus, we perform regression analysis by virtually considering either 10 tissues or 10 treatments as 10 classes, each of which includes 10 replicates. Table 1 is the confusion matrix between genes associated with significant adjusted P-values (by BH criterion) and the first 1000 genes that belong to one of the 10 gene sets. The performances are fairly poor. This is understandable because we forced ten tissues or ten treatments to be ten classes. Interestingly, because the AUC is 0.94 when the treatments are considered classes, the discrimination is successful, although we do not have the means to recognize

Table 1: Confusion matrix of synthetic data using categorical regression. Bold numbers are true positives.

classes = treatments, AUC=0.94						
adjusted P-values	$P < 0.01$	$P > 0.01$	$P < 0.05$	$P > 0.05$	$P < 0.1$	$P > 0.1$
$i \leq 1000$	**2**	998	**16**	984	**77**	923
$i > 1000$	0	29000	0	29000	7	28993
classes = tissues , AUC=0.58						
adjusted P-values	$P < 0.01$	$P > 0.01$	$P < 0.05$	$P > 0.05$	$P < 0.1$	$P > 0.1$
$i \leq 1000$	**0**	1000	**0**	1000	**0**	1000
$i > 1000$	0	29000	0	29000	0	29000

its success without knowledge of the true answer because they are not significant. In conclusion, categorical regression cannot compete with TD-based unsupervised FE.

1.2 SAM

SAM is the next alternative method applied to the synthetic data set. SAM was performed by the sam function in the siggenes package in R (Table 2). Because $p0$, which takes 0.97 (for treatments as classes) or 1.0 (for tissues as

Table 2: Results by SAM. p0 is the ratio of the null hypothesis, and FDR corresponds to the adjusted P-values. Called is the number of genes that break the null hypothesis. Expected number of false positives is False $\times$ FDR $\times$ p0.

classes =treatments , AUC=0.94						classes = tissues, AUC=0.58					
	Delta	p0	False	Called	FDR		Delta	p0	False	Called	FDR
1	0.1	0.974	365.47	799	0.44560	1	0.1	1	0	0	0
2	0.2	0.974	38.59	196	0.19180	2	0.1	1	0	0	0
3	0.3	0.974	2.59	34	0.07421	3	0.1	1	0	0	0
4	0.4	0.974	0.02	3	0.00649	4	0.1	1	0	0	0
5	0.5	0.974	0.02	3	0.00649	5	0.1	1	0	0	0
6	0.6	0.974	0	2	0	6	0.1	1	0	0	0
7	0.7	0.974	0	0	0	7	0.1	1	0	0	0
8	0.8	0.974	0	0	0	8	0.1	1	0	0	0
9	0.9	0.974	0	0	0	9	0.1	1	0	0	0
10	1.0	0.974	0	0	0	10	0.1	1	0	0	0

classes), corresponds to the ratio where the null hypothesis is true, basically no significant distinction between classes is detected by SAM. In contrast, the AUC is 0.94 when the treatments are assumed to be classes; again, the

discrimination is successful, although we cannot recognize its success without the knowledge of the true answers because they are not sufficiently significant (i.e., FDR is not small enough to be significant).

1.3 limma

limma is yet another method to identify genes that are expressed differentially between multiple classes. limma was performed using the limma package in R. Here is an R code to perform limma. For treatments as classes,

```
design <- model.matrix(~0+factor(rep(1:10,each=10)))
colnames(design) <- 1:10
fit <- lmFit(x_all, design)
fit <- eBayes(fit)
```

For tissues as classes,

```
design <- model.matrix(~0+factor(rep(1:10,10)))
colnames(design) <- 1:10
fit <- lmFit(x_all, design)
fit <- eBayes(fit)
```

where matrix x_all include x_{ij}. Although limma should be applied to a logarithmic ratio because synthetic x_{ij} includes negative values, we considered that x_{ij} has already been converted to a logarithmic ratio. Table 3 shows

Table 3: Confusion matrix of synthetic data using limma. Bold numbers are true positives.

classes = treatments, AUC=0.99

adjusted P-values	$P < 0.01$	$P > 0.01$	$P < 0.05$	$P > 0.05$	$P < 0.1$	$P > 0.1$
$i \leq 1000$	**2**	998	**10**	984	**82**	923
$i > 1000$	0	29000	0	29000	0	29000

classes = tissues, AUC=0.99

adjusted P-values	$P < 0.01$	$P > 0.01$	$P < 0.05$	$P > 0.05$	$P < 0.1$	$P > 0.1$
$i \leq 1000$	**0**	1000	**0**	1000	**0**	1000
$i > 1000$	0	29000	0	29000	0	29000

the confusion matrix obtained by limma. Because AUC is 0.99 for both tissues as classes and treatments as classes, limma is better than the above two methods, although we could not obtain significant results for the 1,000 genes that belong to the 10 gene sets because of the lack of ability to find the significance if we do not know the real answer.

1.4 Multi-view bi-clustering

Multi-view bi-clustering (MVBC) is a new method that can recognize bi-clustering integrating multiple observations. As seen in Fig. 1, x_{ij} is a block diagonal. Thus, bi-clustering may be possible. We applied MVBC using the

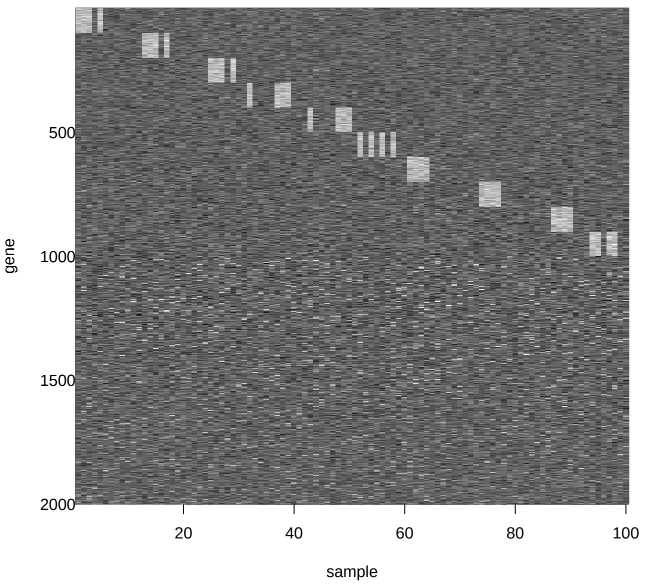

Figure 1: Heatmap of first 2000 genes. Brighter indicates more expressive.

mvlrrl0 function in the mvcluster package in R, regarding 10 treatments as 10 views. mvlrrl0 is the multi-view bi-clustering considering sparseness. Thus, it is suitable to identify a small number of expressive genes ($\sim$ 1000) separated from many non-expressive genes ($\sim$ 29000). The maximum numbers of genes and tissues to be selected in each view were set to 1,000 and 4, respectively, because there are 1,000 expressive genes in any of the classes and 4 tissues for each treatment that are expressive in the present synthetic data. maxIter is set to 10,000 because it did not converge in the default setting. Table 4 shows the results. This suggested that even when bi-clustering is used that

Table 4: Rows: true classification, columns: predicted cluster

	selected	not selected
$i \leq 1000$	**77**	923
$i > 1000$	923	28077

considers sparseness, 1,000 genes are not correctly identified.

1.5 Conclusions

It was obvious that no other methods tested here could successfully process this synthetic data set. Although limma could correctly “rank” genes because no significant P-values are identified, if we do not know the real answer, we cannot trust these results. In contrast to these unsuccessful methods, TD-based unsupervised FE could identify more than half of the 1,000 genes with reasonable adjusted P-values ($P = 0.1$). The failures of the other methods tested here clearly originated in the small number of positives compared with the large number negatives. Because less than 5 % is positive, it is difficult to exclude the effect from the majority of non-critical genes (noise). In contrast, TD is known to work in an unsupervised manner. One thousand genes are placed far from the origin around which the remaining 29,000 genes concentrate. TD-based unsupervised FE is very effective if there are very few positives.

2 Real data set

2.1 Categorical regression

CR was applied to a real data set. The classes are 80 combinations of 10 tissues, 4 treatments and controls or treated samples. The gene expression was standardized within each sample. One may wonder if we should consider only 40 combinations of 4 treatments and 10 tissues by taking the ratio of controls and treated samples prior to CR. However, this experiment is not a matched one; there is no one-to-one correspondence between the treated samples and controls. Thus, we do not take a ratio prior to CR. A more advanced method, e.g., limma, can address this possibility (see below). In contrast to the application to synthetic data, CR can recognize many genes (probes) associated with significant class-dependent expression (Table 5). In actuality, most genes are judged to be expressive in a class-dependent manner. Apparently, it is useful, but does it make sense? Of course, the fact

Table 5: Results of gene selection based on CR.

adjusted P-values	$P > 0.01$	$P < 0.01$	$P > 0.05$	$P < 0.05$	$P > 0.1$	$P < 0.1$
	2222	41157	1986	41713	1839	41860

that almost all genes are expressive in a class-dependent manner is useful and important information. However, the purpose of the present research is to identify candidate genes that induce PTSD-mediated heart disease. To screen the genes further, we need to know what types of regression coefficients are likely to be related to PTSD-mediated heart disease causing genes. In this regard, in spite of the apparent success, CR cannot work along the line we propose.

2.2 SAM

SAM was applied to a real data set with regarding 80 combinations as classes (Table 6). Gene expression was standardized within each sample. Again,

Table 6: Results by SAM. p0 is the ratio of the null hypothesis, FDR corresponds to the adjusted P-values. Called is the number of genes that break the null hypothesis. Expected number of false positives is False $\times$ FDR $\times$ p0.

	Delta	p0	False	Called	FDR
1	0.1	0.011	38538.08	43379	0.0094
2	11.4	0.011	0.02	5424	3.9e-08
3	22.7	0.011	0	323	0
4	34.0	0.011	0	40	0
5	45.2	0.011	0	7	0
6	56.5	0.011	0	4	0
7	67.8	0.011	0	2	0
8	79.1	0.011	0	1	0
9	90.3	0.011	0	1	0
10	101.6	0.011	0	1	0

SAM identified almost all of the genes as being associated with some significant differential expression. Thus, it has the same problem that CR has.

2.3 limma

In contrast to the above two primitive methods, the advanced method, limma, can specifically consider a differential expression between the controls and treated samples as well. The R code for limma is

When not specifically considering a differential expression between the controls and treated samples,

```
design <- model.matrix(~0+class0)
```

```
colnames(design) <- c(paste("C",1:40,sep=""),paste("S",1:40,sep=""))
fit <- lmFit(log(xr_all[,-1]), design)
fit <- eBayes(fit)
```

and when specifically considering the differential expression between controls and treated samples (case A), following the above,

```
contrast.matrix <- makeContrasts(
C1-S1, C2-S2, C3-S3, C4-S4, C5-S5, C6-S6, C7-S7, C8-S8,
C9-S9, C10-S10, C11-S11, C12-S12, C13-S13, C14-S14, C15-S15,
C16-S16, C17-S17, C18-S18, C19-S19, C20-S20, C21-S21, C22-S22,
C23-S23, C24-S24, C25-S25, C26-S26, C27-S27, C28-S28, C29-S29,
C30-S30, C31-S31, C32-S32, C33-S33, C34-S34, C35-S35, C36-S36,
C37-S37, C38-S38, C39-S39, C40-S40, levels=design)
fit2 <- contrasts.fit(fit, contrast.matrix)
fit2 <- eBayes(fit2)
```

where class0 is a vector having 80 class labels corresponding to the combination of 10 tissues, 4 treatments and controls or treated samples (case B) and xr_{all} includes x_{ij}. Table 7 shows the results of limma. At least for case B,

Table 7: Results of gene selection based on CR.

adjusted	case A : not considering differential expression					
P-values	$P > 0.01$	$P < 0.01$	$P > 0.05$	$P < 0.05$	$P > 0.1$	$P < 0.1$
	0	43379	0	43379	0	43379
adjusted	case B: considering differential expression					
P-values	$P > 0.01$	$P < 0.01$	$P > 0.05$	$P < 0.05$	$P > 0.1$	$P < 0.1$
	25992	17387	17745	25634	13542	29837

specifically considering the differential expression between the controls and treated samples, the situation was improved. The selected genes are not the most numerous, and they even represent less than half of the set ($P < 0.01$). However, it is still too large to be considered candidate genes that induce PTSD-mediated heart disease.

2.4 Conclusions

In contrast to the application of the synthetic data set, the methods tested in this supplementary document identified too many genes. Because the purpose of this study was to identify candidate genes that induce PTSD-mediated heart disease, the identification of more than ten thousand genes is useless.

In actuality, the identification of too many positives occurred because of the large samples (in total, more than a few hundreds). In this case, very small differences are considered critical, but it does not have any biological meaning.

3 Conclusions of comparisons with other methods

The other methods tested here exhibited contrasting tendencies between the application to synthetic data and that to a real data set. When they are applied to a synthetic data set, they can detect too small a number of genes, whereas almost all of the genes were identified when they were applied to a real data set. It is very different from the successful identification of a limited number of genes by TD-based unsupervised FE when applied to the synthetic data as well as real data. This difference possibly comes from the fact that TD-based unsupervised FE tries to evaluate individual genes in contrast to the whole gene set because outliers were selected. However, the other methodologies evaluate genes based on absolute P-values. Because of its very distinct strategy, TD-based unsupervised FE is a very promising method that is applicable to a wide range of bioinformatic analysis.
